# What makes you different? A qualitative study on experiences of not fitting in with social norms across cultures

**DOI:** 10.1186/s40359-026-05130-z

**Published:** 2026-07-11

**Authors:** Luzia C. Heu, Adebunmi Oyekola, Naveli Sharma, Andrés E. Montiel, İlayda Piri, Hodar Lam

**Affiliations:** 1 Department of Interdisciplinary Social Science, Utrecht University, Utrecht, Netherlands; 2 Department of Counselling and Human Development Studies, University of Ibadan, Ibadan, Nigeria; 3 Department of Management Studies, Delhi School of Business (VIPS-TC), New Delhi, India; 4 Department of Psychology, Lingnan University, Tuen Mun, Hong Kong, China

**Keywords:** Social norms, Norm deviations, Loneliness, Culture, Social exclusion

## Abstract

People who deviate from social norms often face negative consequences such as exclusion, loneliness, or impaired wellbeing. While previous research has focused on consequences for specific minority groups, we know little about experiences of deviating from the norm in the general population, and about consequences and remedies that people perceive thereof. To fill these gaps, we conducted 62 qualitative in-depth interviews with young adults ages 18–30 years --- an age group that seems relatively sensitive to norm deviations. As social norms and sanctions vary cross-culturally, we sampled from different countries, including Nigeria, India, Turkey, South China and Hong Kong, and the Netherlands. Across cultural samples, participants reported a wide range of perceived norm deviations (e.g., in preferences; developmental timing; family relationships). A large share reported perceiving to deviate *recurrently* despite not being part of a defined minority – for instance, due to introversion or earlier experiences of not fitting in (e.g., with family; classmates). Numerous negative reported consequences (e.g., insecurity; social withdrawal; exclusion) suggest the high relevance of not fitting social norms for social and mental wellbeing. Specifically, interventions may need to target experiences of deviation outside minority groups more (e.g., through support groups; psychotherapy). Possible cultural differences are discussed.

**Supplementary Information:**

The online version contains supplementary material available at https://doi.org/10.1186/s40359-026-05130-z.

Social norms facilitate collective living by coordinating individual actions. However, people who do not fit in with social norms often face social sanctions, unfavorable self-perceptions, and loneliness [36–38]. As such, experiences of not fitting in with social norms may be a relevant risk factor for impaired social and mental well-being, and, accordingly, for impaired health more generally (e.g., [13, 27, 46, 51]. While previous studies have researched individuals because they were part of defined marginalized groups (e.g., sexual minorities, [19], there has been little exploration of not fitting in with others in the general population [35].

In the current qualitative study, we thus aimed to examine which social norms young adults perceive to deviate from, which consequences of not fitting in they perceived, coping strategies, and suggestions for intervention. As social norms vary across cultural contexts (e.g., [14, 37]), we conducted and compared in-depth interviews from Turkey, Nigeria, India, South China and Hong Kong, and the Netherlands. We also explored differences between situational experiences of not fitting in and recurrent experiences (i.e., feeling different across various social contexts and for many years). After all, it is mainly recurrent experiences that require intervention. This research may help deepen our understanding of what norm deviations in the general population look like in practice, and which social norm deviations require more attention in future studies and practice. In addition, it may offer starting points for the development of interventions to support people who suffer from being or feeling ‘different’.

## Social norms, not fitting in and its consequences

Social norms coordinate individual behavior in groups, and have been suggested to maintain social order [69]. As such, they facilitate collective co-existence and reduce the number of decisions individuals need to make in their (daily) lives (e.g., about how to greet others; whether to move out when of age). Social norms can be defined as unwritten rules about what most members of a group are like or do (*descriptive* norms), or about what they ought to do (*injunctive* or *prescriptive* norms; [15]). In practice, these different types of social norms are not clearly distinguished by lay people [21], and will thus also not be distinguished in this paper. Social norms permeate most – if not all – areas of life, but the relatively few existing taxonomies of social norms have, for instance, described social norms about friendship [3, 24, 25] or norms that are specific to a certain geographical context (e.g., UK, [26]).

Social norms can influence individual behavior and thought irrespective of whether they reflect actual shared group characteristics or not. People seem to often strive to conform to social norms that hardly any group member privately holds (i.e., *pluralistic ignorance*; [2]). For instance, US American college students overestimated how comfortable other college students felt with drinking much alcohol, which changed their personal attitudes toward alcohol and led to more alcohol consumption over time [60]. Similarly, people have been found to sometimes agree with others on obviously wrong judgments (e.g., about the length of two lines in the famous Asch experiments, [5, 8]). This is why it is relevant to not only study actual norms, but particularly also *perceptions* of social norms.

Despite the influence of social norms on human behavior, not everyone adheres to them. While some research has focused on active behavioral ‘violations’ of social norms [69], people usually do not *voluntarily* deviate. Nevertheless, non-conformity seems to be met with sanctions around the globe [22]. People who visibly do not adhere to social norms are more likely to be met with open confrontation, anger or blame, social exclusion, rejection, or gossip (e.g., [22, 37, 69, 71] – although some types of sanctions are more common in some cultures than others. Even deviations that are invisible to others can have negative consequences: People who feel that they do not fit in with norms are more likely to feel misunderstood by others, experience shame, guilt, or regret, have lower self-worth, or avoid close relationships [35, 37, 69]). Against this backdrop, it is unsurprising that people who deviate from social norms seem to experience more or longer-lasting feelings of loneliness than people who conform [10, 32, 36, 50, 63].

Defined minority groups such as cultural, ethnic, sexual, gender, or neurodivergent minorities, or people with chronic illness or disability are more likely to experience not fitting social norms than the general population. Accordingly, most scientific information on consequences of ‘being different’ seems to stem from research on minorities [18, 19, 32, 48, 57, 58, 61, 73]. Indeed, members of minority groups seem to experience overall poorer health and lower well-being than the general population [39, 47, 55]. According to minority stress theory, these disparities can be explained by the added stress that stigma against minorities generates. For instance, stigma manifests through discriminatory policies and laws, everyday experiences of discrimination (e.g., disrespect by others), internalized stigma (e.g., self-rejection), or concealment of personal tendencies or part of one’s identity [27, 55, 61]. Additionally, such disparities may also be explained by social issues such as ostracism, social isolation, or loneliness, which are relevant risk factors for poorer health (e.g., [13, 46]).

Less research has engaged with experiences of deviating from social norms in the general population. Such research can mainly be found in the management literature, focusing on experiences of not fitting in at work as person-environment (mis)fit or employee misfit in organizations [17, 67]. In a qualitative study, employees perceived not fitting in with others on sociodemographic characteristics such as gender, sexuality, age, but also length of tenure [17]. Other individual characteristics that employees perceived to deviate from were personality characteristics, cognitive style (i.e., thinking differently than others), values, or desire for social interaction at work. The authors also identified factors contributing to feelings of not fitting in. These included few social opportunities in the workplace, existence of social cliques, having a different type of employment contract than others, or working in roles that involved separation from them (e.g., being in a leading position; working on a separate topic). In a quantitative study, employees at a Dutch public service organization most frequently reported perceiving to be dissimilar from others in personality, followed by ethnicity/culture, age, work experience, religion, sexual orientation, disability, education level, political orientation and gender [65]. On more than half of these dimensions, perceived dissimilarity was associated with less perceived inclusion. Overall, however, there has been little empirical examination of which social norms people in the general population typically perceive to deviate from, and which consequences they experience themselves --- a gap we aim to fill through the current study.

### Situational versus recurrent experiences of not fitting in

While most people perceive to deviate from norms in concrete situations (e.g., at a party), others do not fit in more systematically or ‘recurrently’ --- for instance, because they deviate from broader cultural or societal norms. They are likely to experience not fitting in across different social contexts and throughout a large share of their lives. While such recurrent experiences of not fitting in are again particularly likely to affect minority groups, the wide range of social norms and hence of possible norm deviations suggests such experiences in other parts of the general population as well [35]. For instance, when we examined self-perceived reasons for loneliness [38], we could observe not fitting gender norms, being less talkative/more introverted than others, or having different education level as norm deviations that people seemed to recurrently struggle with. Understanding differences between recurrent and situational experiences of not fitting in may be relevant for the development of interventions, since situational experiences are arguably an inherent part of life, while recurrent experiences may cause considerable suffering and signal a need for change.

While recurrent perceptions not to fit in can be rooted in actual differences from others, they may also result from heightened sensitivity to not fitting in or to being rejected (*rejection sensitivity*; [70]). For instance, people who were excluded or rejected earlier in life have been suggested to become more alert to being rejected, and more likely to interpret ambiguous situations as rejection. To protect themselves, they may then act in more hostile or aggressive ways, feeding the self-fulfilling prophecy that others withdraw from them. While such rejection may happen with peers (e.g., at school), psychotherapy research and attachment theory typically attribute adult relational patterns and emotions to early family dynamics [56]. Indeed, in a longitudinal study following 227 US American families, higher (verusus lower) rejection sensitivity in adolescence was preceded by more maternal harshness, more maternal control, less predictability of nurturance, or more family conflict earlier in childhood [30]. To better understand the experiences and needs of people who might need future intervention --- arguably particularly people who perceive to deviate recurrently --- we explored differences in experiences of people with situational versus recurrent experiences of not fitting in.

### Cultural variation

Social norms and the extent to which adhering to them is socially expected vary by culture. Cultures are defined by ideas that are imperfectly shared by members of a group, including social norms [14]. For instance, premarital romantic relationships or physical intimacy tend to be normative in cultures with less traditional family values yet they can be strongly stigmatized in cultures with more traditional family values (e.g., [4]). Accordingly, being single in adolescence or young adulthood is likely to be perceived as a stronger norm deviation in cultures with less (versus more) traditional family values, with more negative consequences such as loneliness [37]. Additionally, in so-called ‘tight’ cultures, conforming to social norms is considered more important, the range of normative characteristics is narrower, and deviation more socially sanctioned than in so-called ‘loose’ cultures [28]. To examine cross-cultural consistency and variability in experiences of deviating from social norms, we sampled from different cultural contexts, including Nigeria, India, Turkey, South China and Hong Kong, and the Netherlands.

### The current research

As social norms vary by age (e.g., [50, 63]), we focus on experiences of not fitting in among young adults ages 18 to 30 years. Adolescents have been found to be relatively sensitive to social rejection, suggesting that, during this developmental period, people may have more experiences of not fitting in and experience more negative consequences for it than other age groups [31]. At least in part, such sensitivity to social rejection is likely to continue into young adulthood [70].

Through qualitatively examining (1) which social norms young adults perceive to deviate from in different cultures and (2) perceived consequences of such norm deviations, we aimed to fill previous theorizing and research about deviating from social norms (e.g., [22, 37]) with real-life examples from different cultural contexts, promoting a deeper understanding of what not fitting in with norms means in practice. Furthermore, by (3) comparing experiences of people with recurrent versus situational experiences, we aimed to explore why some people keep experiencing that they do not fit in --- to better cater to their experiences and needs in future interventions. Relatedly, by (4) collecting coping strategies and suggestions for intervention by people with lived experiences, we aimed to offer possible starting points for how to support people who recurrently feel that they do not fit in.

## Method

### Sample selection

After a pilot study with participants from various cultural contexts, we aimed to sample from cultural contexts that would differ in the content of social norms and social importance attached to adhering to social norms. For one, we thus sampled from countries that differed in the extent to which traditional family values or traditional gender roles were endorsed, based on data from Wave 6 (2010–2014; [40]) and Wave 7 (2017–2022, [34]) of the World Values Survey (see Table S1 in the supplementary materials). These are clusters of social norms that tend to differ across cultural contexts. Furthermore, we sampled from countries that had been found to have different levels of tightness-looseness (i.e., how strongly human behavior is regulated by social norms; how strictly adhering to such social norms is sanctioned; [22, 29, 68]). The final selection of cultural contexts also depended on the availability of collaborators and/or research assistants to adapt the research to the respective cultural context, conduct it in the local language, as well as translate and interpret transcripts. Contexts we eventually sampled from varied considerably on the selected cultural characteristics, as displayed in Table S1.

### Recruitment and sample

We used a mix of purposive and convenience sampling, aiming to interview individuals between the ages of 18 and 30 years, of different gender and education levels, including individuals with and without minority status (e.g., sexual or ethnic minorities). In each context, participants were invited to participate in a study about not fitting in, and recruited from extended social networks of researchers involved in the project, through social media, and at a workshop about loneliness in the Dutch context. In line with Heu, Hansen, et al. [38], we aimed for 6–10 interviews per context. While theoretical saturation would be desirable, it is difficult to achieve for a broad topic like deviations from social norms. Theoretical saturation was reached for perceived consequences of not fitting in.

We first conducted a pilot study with 12 participants from different countries (e.g., Venezuela, Colombia, the UK, Poland, Austria, Germany). Most participants in this international sample were or had been living abroad at the time of the interview, and were interviewed in English (except for participants from Austria, who were interviewed in German). As interviews from the pilot study resulted in valuable data and materials were not considerably changed afterwards, these interviews were analyzed together with subsequently collected data as ‘international sample’.

The final sample then consisted of 62 interviews, conducted between 2022 and 2025: Additional to the international sample from the pilot study, we interviewed 10 young adults in Nigeria, 10 in India, 13 in South China and Hong Kong, 11 in Turkey, and 6 in the Netherlands. These interviews were conducted in English or Yoruba in Ibadan and surroundings, Nigeria; in English, Hindi, or Gujarati in Delhi and Gujarat, India; in Turkish in Turkey; in Mandarin or Cantonese in South China/Hong Kong; and in Dutch in the Netherlands. Two interviews with Dutch participants living in the Netherlands, and one interview with a Chinese participant living in the Netherlands were initially conducted as part of the pilot study but later analyzed as part of the Dutch and Chinese/Hong Kong samples. As such, these interviews were conducted in English. For sample characteristics, see Table 1.

**Table 1:** Sample characteristics

	International sample (*n* = 12)	Nigerian sample (*n* = 10)	Indian sample (*n* = 10^a^)	Chinese and Hong Kong sample (*n* = 13)	Turkish sample (*n* = 11)	Dutch sample (*n* = 6)
Age range	22–29	19–30	20–30	19–29	19–28	21–26
Gender						
Men	3	5	4	6	3	4
Women	9	5	6	7	8	1
Non-binary	-	-	-	-	-	1
Education level						
Secondary school education	2	5	-	1	-	3
Vocational training	1	1	-	1	2	-
Undergraduate university studies (bachelor studies)	6	3	6	3	7	1
Graduate university education (master or PhD studies)	3	1	4	8	3	2
In a romantic partnership						
No	4	6	9	9	5	5
Yes	8	4	1	4	7	1
Type of feeling different						
None or situational	5	5	1	8	2	2
Recurrent	6	4	8	4	6	4
Recurrent, but not throughout life	1	1	1	1	3	0

The international sample consisted of 3 participants from Venezuela, 1 from Colombia, 3 from Austria, 2 from Germany, 1 from the UK, 1 from Poland, and 1 from Belgium/the US

^a^The research assistant in Gujarat, India, could not be reached anymore after conducting a first interview. Accordingly, only one interview is from Gujarat, while other interviews are from Delhi. For the Gujarati participant, data on age was missing. As the interview was otherwise conscientiously conducted, it was included in the dataset

### Procedure

Before participating in in-depth interviews, participants filled in a brief online survey for informed consent and key sociodemographic data (e.g., age, gender). In contexts where an online survey was less suitable (e.g., Nigeria), informed consent was collected on paper and sociodemographic information during the interview. In-depth interviews could either be conducted online or in person, depending on the participant’s location and preference.

During semi-structured in-depth interviews, we asked about examples of situations in which people felt like they did not fit in with others or were different from others, which norms they deviated from (e.g., *How were you different from others? What did others around you do that you did not do?*), and how often they experienced not fitting in with others. We also asked about emotions, thoughts, or other consequences that were elicited by perceptions of being different (e.g., *How did this make you feel? What kinds of thoughts came up in that situation?*) and how participants felt better (e.g., *When and how did you not feel different anymore? Can you think of anything that would have helped you in that situation?*). Finally, we collected participants’ ideas about interventions (e.g., *Do you have any ideas about how people who repeatedly experience situations like this* [not fitting in] *could be supported*?). The full interview guide can be found on the Open Science Framework (osf.io/mz4wk).

Notably, except for in the pilot study, all interviews included a module about loneliness. Although answers to the questions about loneliness were not aimed at providing data for the current study, participants often provided information about not fitting in when talking about loneliness, and vice versa. This is unsurprising as loneliness can result from feeling different from others [36, 38]. Consequently, some information presented in this paper was elicited by questions about loneliness.

### Data preparation and analytical approach

All interviews were transcribed (e.g., either manually or through software such as Amberscript) and translated into English by collaborators, research assistants, or LH (unless interviews were conducted in English). Interviews were checked by LH, and questions about translations or interpretations discussed with collaborators or, if necessary, additional translators.

Interviews were then thematically analyzed [9] by LH, using predominantly inductive coding. Codes were mainly based on what participants explicitly reported (i.e., a semantic approach), and occasionally on concepts or assumptions driving their narratives (i.e., a latent approach). Codes were developed to describe norm content, consequences of deviating from norms, as well as coping strategies and perceived remedies. Although not explicitly asked for it, participants also often reported causes for not fitting in (i.e., characteristics increasing the likelihood for a person to perceive not to fit in), which were coded as well.

**Fig. 1:**
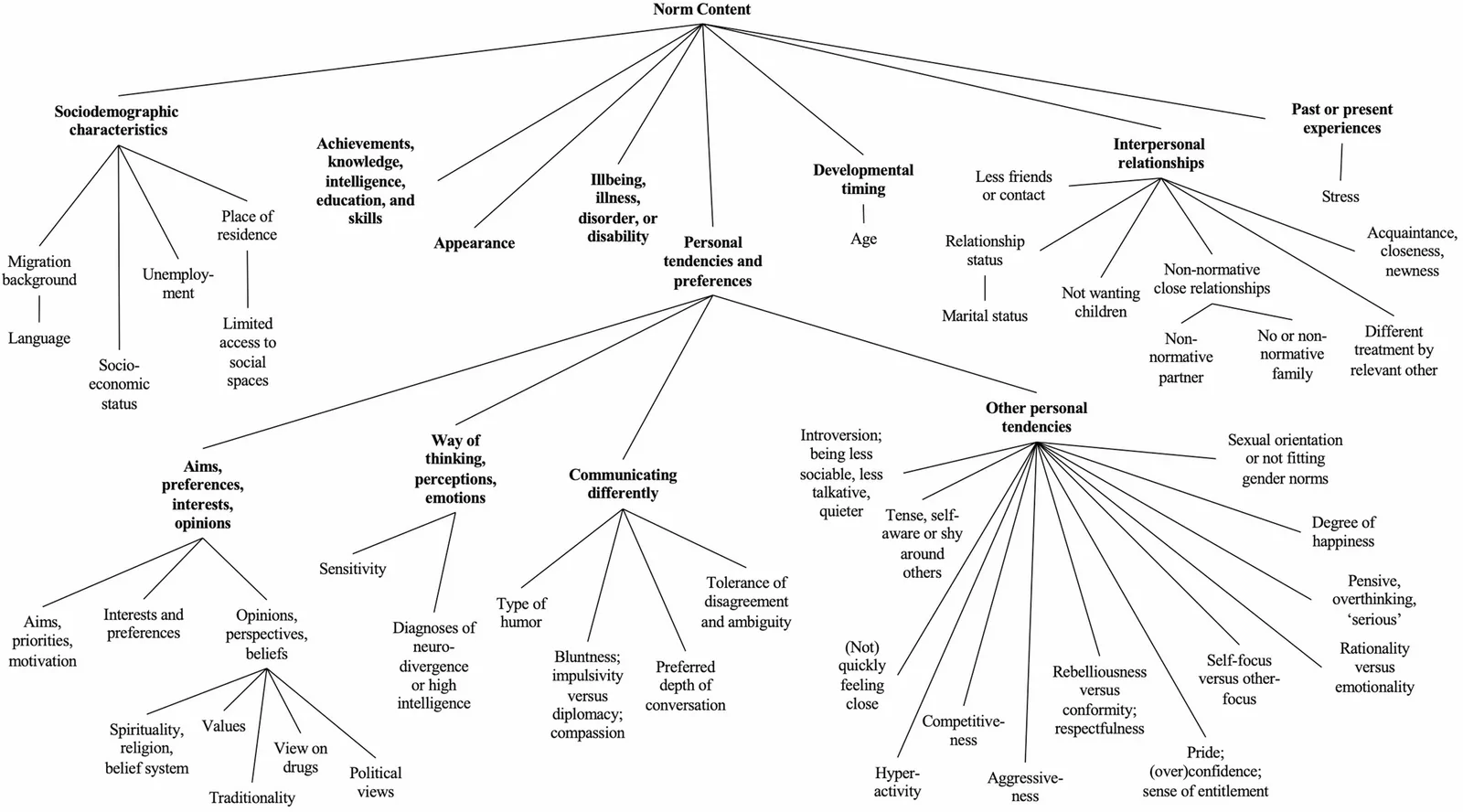
Social norms that participants perceived to deviate from

**Fig. 2:**
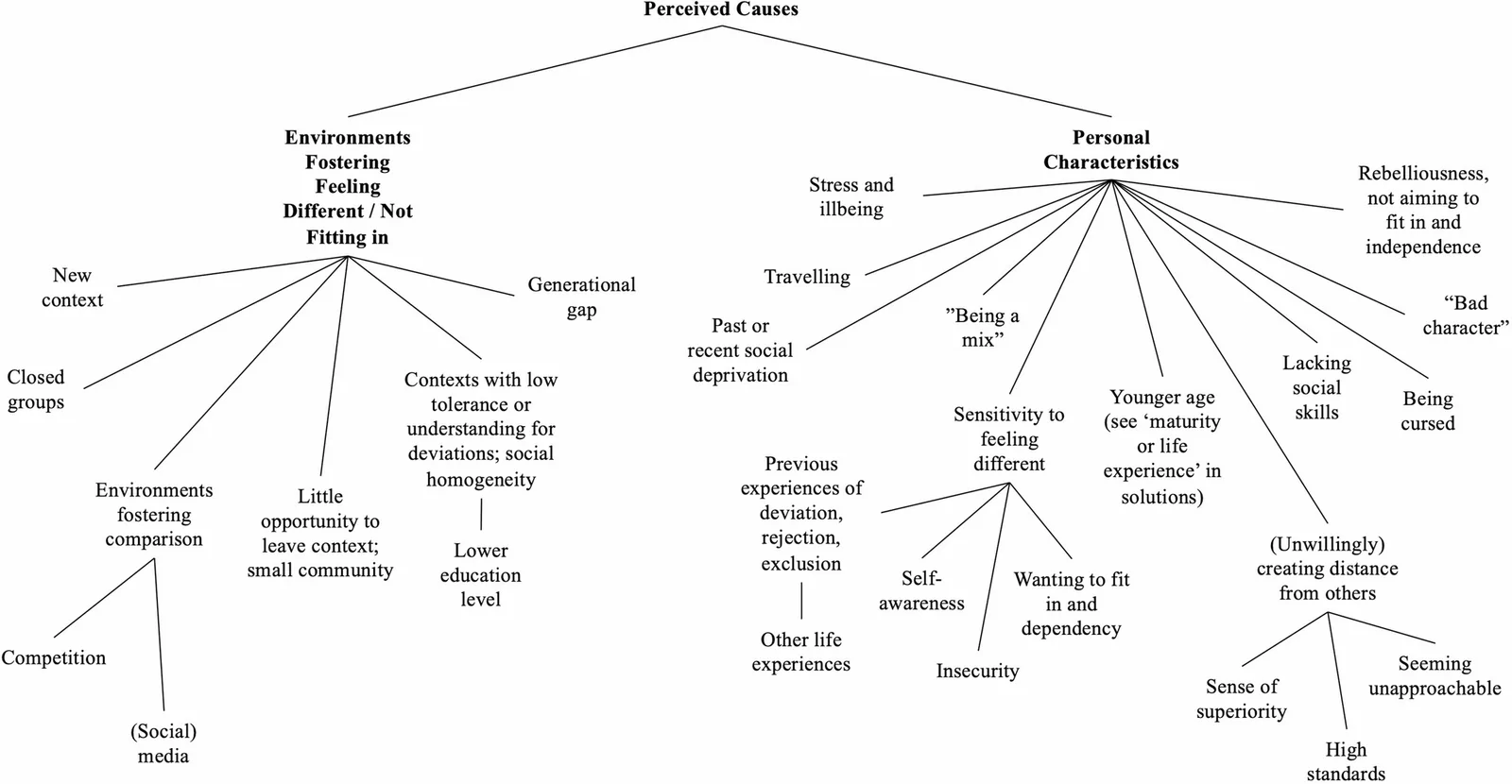
Perceived causes for a higher likelihood to experience not fitting in with social norms

**Fig. 3:**
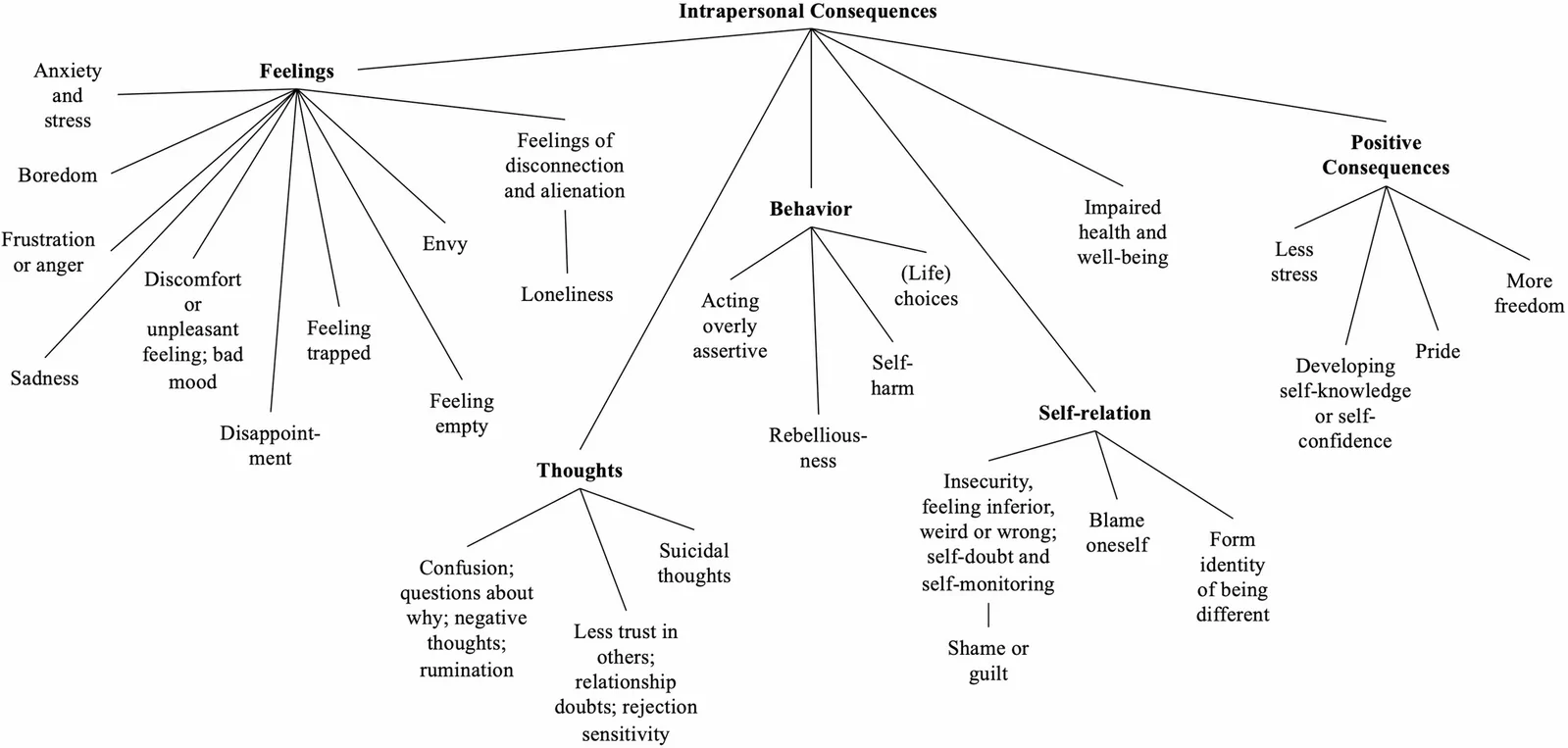
Perceived intrapersonal consequences of not fitting in with social norms

**Fig. 4:**
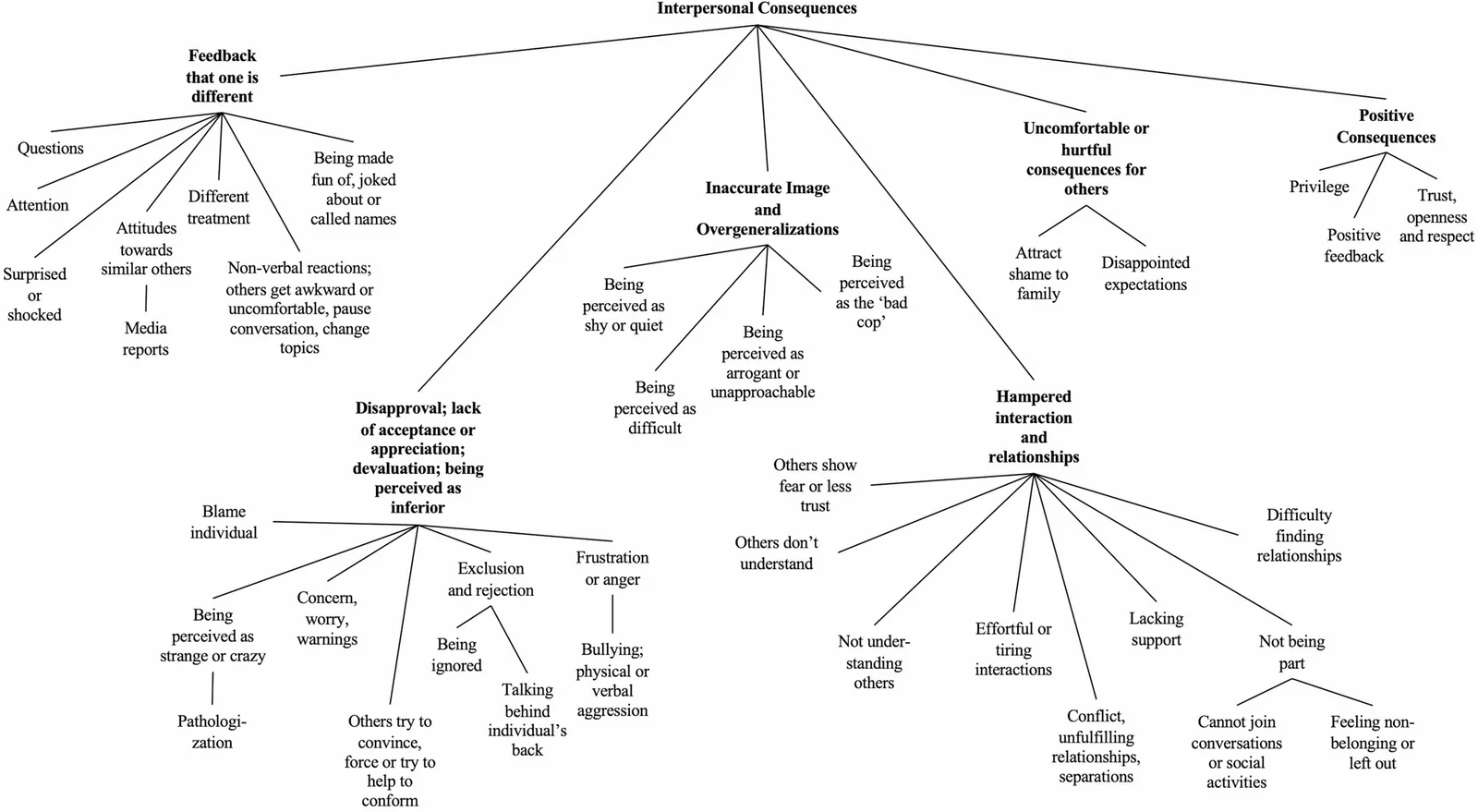
Perceived interpersonal consequences of not fitting in with social norms

**Fig. 5:**
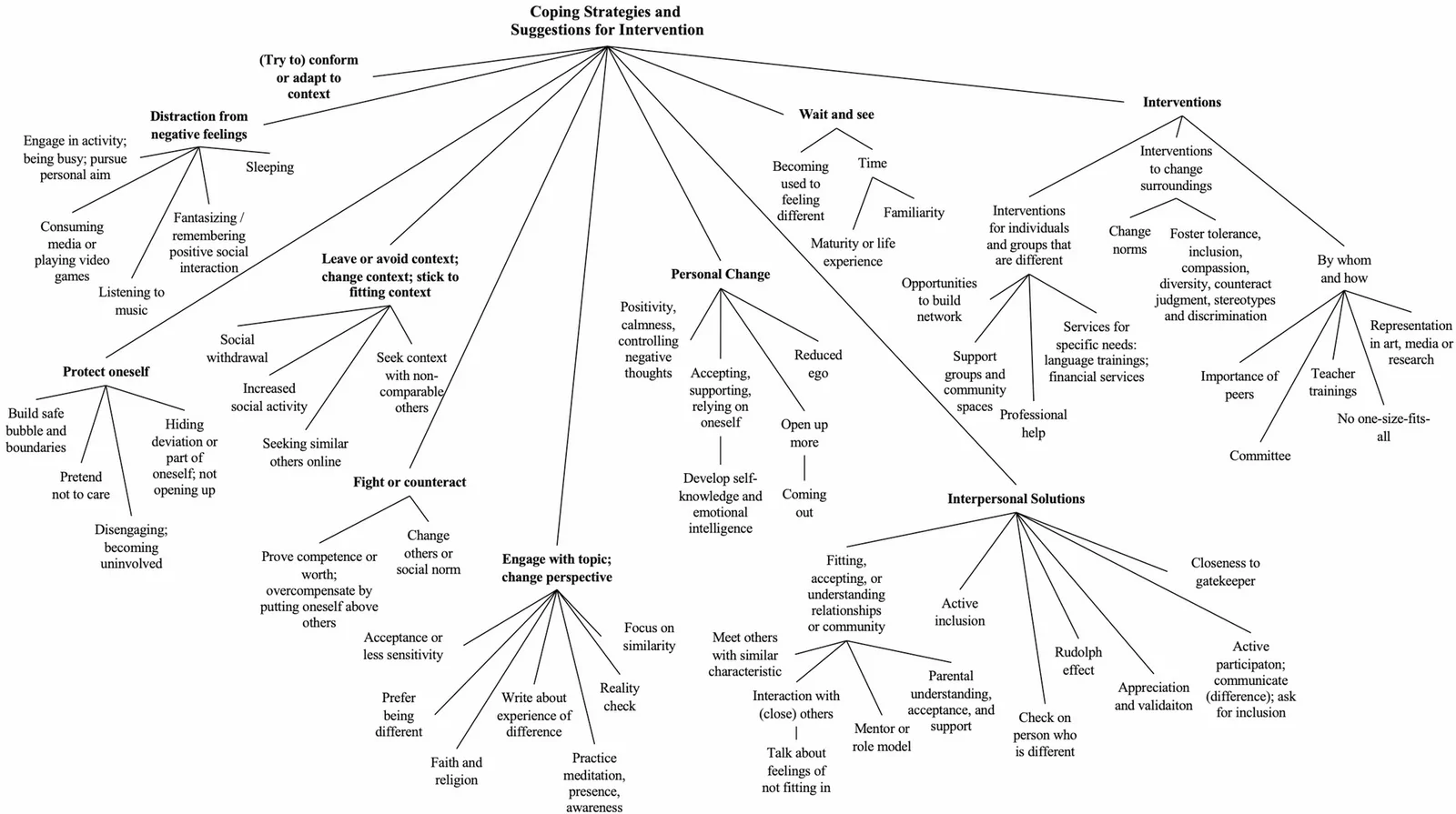
Reported coping strategies and suggestions for intervention

Throughout the analysis, codes were continuously revised and organised. Similar or related codes were combined, more detailed codes were developed (e.g., if multiple participants mentioned the same sub-aspect of a higher-order code; if a theme only emerged in one cultural sample), and codes were summarised under different themes. The final coding trees are displayed in Figs. 1, 2, 3, 4, 5 and quotes for each point in this paper are presented in Tables S2.1-S2.5 in the supplemental materials.

Lastly, occurrence and content of codes were compared across different cultural groups and between groups with recurrent versus situational experiences of not fitting in. Interviews were categorized as instances of recurrent experience of not fitting in with social norms if participants reported having felt different for the larger share of their lives and/or across various different social situations (e.g., in family, with colleagues, and with friends). If participants reported only concrete situations or concrete social contexts in which they did not fit in (e.g., in a specific team; with their partner’s friends; during their master’s program), their interviews were categorised as instances of situational experiences. Seven participants reported experiences that fell somewhere in between and were summarised into a third group with ‘recurrent experiences that do not last throughout life’. Given the small sample size in this category, comparisons were mainly made between groups with situational and recurrent experiences. All interviews were categorized by LH. A justification of each categorization was documented in a table, together with a summary of each case and example quotes. This table was shared with collaborators for validation, but not made publicly available to preserve participants’ anonymity. Information on categorizations is available on request. Additionally, NS and AO also categorized interviews from the contexts in which they had interviewed for comparison. Their categorizations converged with those of LH.

### Positionality

Interviews in the pilot study were conducted by AM, a Venezuelan queer man who was a master student at Utrecht University, the Netherlands, and by LH, an Austrian woman working as an assistant professor at Utrecht University. Interviews in Nigeria were conducted by AO, a female senior lecturer, together with a female PhD student and a female master student at the University of Ibadan. Interviews in India were conducted by NS, a female psychology master’s student at the University of Delhi; interviews in South China and Hong Kong by HL, a male assistant professor at Lingnan University in Hong Kong; interviews in Turkey by IP, a female research master student at Utrecht University; and interviews in the Netherlands by LH (see above for characteristics).

Interviews were thematically analyzed by LH, which allowed for consistency in coding across samples. However, interviews were thus widely analyzed in English, rather than in their original language, and coding may be shaped by LH’s cultural lens as an Austrian woman and loneliness researcher. To reduce this influence, translations and interpretations of interviews were discussed with collaborators from each context. Furthermore, coding and conclusions were shared with collaborators for corrections and feedback. There were no disagreements in broader conclusions drawn from the data.

## Results

### Types of social norms that participants perceived to deviate from

In line with previous literature examining experiences of stigma and exclusion in minority groups, some participants reported not fitting in due to physical disability (*n* = 1) or mental disorder (*n* = 2; e.g., [48]), ethnicity (*n* = 1, e.g., [57]), sexual orientation (*n* = 8, e.g., [19, 73]), unconventional gender expression (*n* = 1, [53, 73]), or neurodivergence (*n* = 2, [32, 61]) (*n* = 12 in total because one participant belonged to different minority groups; see Table S2 for quotes). Additionally, 8 participants reported having felt different when temporarily moving to a different context (i.e., temporarily belonging to a cultural, ethnic, or religious minority; e.g., [18, 58]). Most of these participants belonged to the international sample (except for one participant from the Chinese, and one participant from the Nigerian sample).

Nevertheless, Fig. 1 illustrates that norms that people perceived to deviate from extended far beyond these few concrete categories. Indeed, 40 participants reported experiences of not fitting in without belonging to a minority, and most participants who did belong to minority groups also reported experiences of not fitting in that were unrelated to their minority status. While some quotes are provided in this Results section, most quotes can be found in Tables S2.1-S2.5. Note that some interviews were conducted in English, and quotes may thus sometimes not be entirely grammatically correct or reflect local accents.

The social norms that people most commonly perceived to deviate from fell under having different preferences and perspectives than others; different achievements or skills (e.g., being highly intelligent; knowing less in a certain area); different personal tendencies or personality characteristics (mainly being less outgoing or sociable); different appearance (e.g., dressing differently; being taller or bigger; being less beautiful); or different developmental timing (e.g., engaging in romantic relationships later; feeling more mature than others). Next to these personal characteristics, participants also reported feeling different for having non-normative relationships or going about their relationships differently than others, such as having no contact with family, having very strict parents, being less acquainted with others, or feeling treated differently by others. Notably, characteristics that made participants feel that they did not fit in could often trigger additional distinguishing characteristics. For instance, having a non-normative family (e.g., an ill parent) could be related to also feeling different from others due to different life experiences, more mental health issues, or different priorities in life.

Notably, participants described deviating from others in diverse contexts, ranging from families to the work setting to society at large. Although not explicitly asked about it, some participants mentioned factors that may increase the likelihood of experiencing feelings of not fitting in with others (see Fig. 2). A relatively common pattern was for participants to report having felt different more when they were younger, and less so at the time of the interview. This supports the notion that fitting in with others becomes particularly relevant in adolescence and decreases in relevance with age [31]. Indeed, multiple participants related less experiences of not fitting in to ‘being more grounded in themselves’, less sensitivity, and being better able to manage their feelings.

I wasn’t a teenager anymore, but I was younger than now and I feel like it’s already a difference that I’m older now. And I feel more grounded cause I’ve had more experiences. […] Like, [some years ago,] I was still, like, more, like, seeking of who I want to be, and what I want to be like. And groups of friends, like, influence that for sure. (PS5, Polish, 29 years old, female, situational)

Such changes were also often related to having more opportunity to choose one’s own social network upon leaving school.

But also, quite simply, I’ve grown older. I’m not obliged to sit in a room with people every day who don’t like me. I just have a bit more freedom to choose my own people. (NL3, Dutch, 22 years, transgender male, recurrent)

According to participants, other situational factors increasing the likelihood of feeling different included being new to a group (in line with [17]) or moving to a different culture.

Participants also discussed environmental causes, such as contexts with little tolerance for deviation, including homogeneous groups or environments with strict or narrow norms, environments that offer few opportunities to leave or choose social contexts (e.g., small schools), or that foster comparison (e.g., competitive environments). Finally, in line with theorizing on rejection sensitivity (e.g., [70], participants perceived that some people are more sensitive to not fitting in than others. They explained such sensitivity by insecurities, high self-awareness, a strong desire to fit in, stress or illbeing. Some of these factors may also explain why some people recurrently experience to be different from others, which will be addressed next.

### Recurrent experiences of not fitting in

Qualitative interview data allow to describe phenomena and study qualitative differences between groups, but frequencies and, relatedly, differences in the occurrence of specific norm deviations (i.e., quantitative differences) need to be interpreted with caution. Consequently, differences in the occurrence of themes between groups will only be discussed if they are striking (e.g., no occurrence among people with situational experiences, yet reported by several or most of participants with recurrent experiences).

More than half of participants reported experiencing not fitting in with others across various social contexts and throughout a large share of their lives (see bottom of Table 1 for proportions; top of Table S2.2 for example quotes). This may be attributed to self-selection arising from the study invitation. Nevertheless, participants’ reports allowed to study particularly those experiences of deviating from social norms that may require intervention due to their wide-reaching and long-lasting consequences.

There were hardly any specific norms that *only* people with recurrent experiences reported to deviate from – that is, experiences that were not at all reported by participants with situational experiences of not fitting in. Nevertheless, some themes seemed to be discussed more by participants with recurrent experiences. In line with previous research, multiple participants reported having felt different from others for a large share of their lives because of being part of a minority. Indeed, 11 out of 12 participants who belonged to a minority group throughout their lives (i.e., not only when they temporarily lived abroad) reported recurrent experiences of not fitting in. Nevertheless, 21 participants with recurrent experiences of not fitting in did not belong to a defined minority, and some members of minority groups reported experiences of not fitting in that were unrelated to their minority status. Some participants reported feeling that they did not fit into their ‘culture’ or ‘society’ because of, for instance, generally different interests, opinions or values (e.g., regarding level of religiosity). Some participants with recurrent experiences reported that their different life experiences (e.g., having lost or cared for a family member) had made them feel different from others.

Several participants attributed their recurrent experiences of being different to not conforming to the norm of being sociable or outgoing --- to being less talkative, quieter, shyer, or more introverted than others.

Due to my shyness and introversion, I was somewhat of a reserved person [during school time], which made me feel like I didn’t fit in with the more energetic and sociable girls, especially in adolescence. That feeling is still there. I feel really strange and uncomfortable when I’m around more active, outgoing, and social people because I can’t be like them. […] For example, it happened when I was with my boyfriend’s friends. They were more like the type I described earlier [energetic, outgoing] and I felt like I couldn’t communicate with them. It felt as if there was a kind of distance between us, even though there wasn’t really. I felt like I was inferior to them. (T6, Turkish, 26 years, female, recurrent)

Multiple participants who reported recurrent experiences of not fitting in mentioned having a different way of thinking, perceiving or feeling than others. Mainly in western samples, participants reported having explored medical diagnoses explaining these experiences (or being sent to mental health professionals by their parents) – for instance, diagnoses of autism, ADHD, or high intelligence.

[…] when I was about 20, I did this, like, assessment with a psychologist, […] and it was for this, what the French call *haut potentiel*, so like high potential [...] And the psychologist that I did it with basically said that I could wear that label. Uhm, and uhm, and then I read a book about that and it helped me understand why I had always felt so different. But then, this summer, actually, I did an assessment with a neuropsychologist here in Brussels. […] he said that there were autistic traits but I don’t know if that means that, like [I have the diagnosis of autism] […] I don’t really know why I feel like I need an official diagnosis because when I read stuff online about autistic women, I really connect with that. (PS12, US American/Belgian, 29 years, female, recurrent)

The quote above illustrates the common search for an explanation for recurrent experiences of feeling different from others. Indeed, multiple participants reported feeling different or being treated differently than others without knowing what for, and ruminating about how and why they felt different (see below for consequences of not fitting in).

I just feel like, if you and I get to the same place now, maybe to make an enquiry, that person is definitely going to warm more with you and like you better than me. It [is] something that happens most time. […] Maybe they think I’m proud. Maybe... I don’t know. […] The thing is people just withdraw from me with no tangible reason(s). At least, give me a reason, let me work on myself, but no. […] I really don’t know what to do. (NG3, Nigerian, 26 years, female, recurrent)

In line with the notion that earlier experiences of rejection can lead to heightened sensitivity to being rejected later in life [30, 70], particularly participants with recurrent experiences mentioned feeling different or being signaled to be different in childhood or adolescence already, for instance in their families or at school.

To the point that during my upbringing, my parents would say: “Well, I think you’re from the milkman, do we still have the receipt? We’ll take you back. I think you were secretly adopted.” Simply because I was very different from my parents when I was younger. For example, my parents are both very extroverted and love to party. And I don’t. […] Um, the realisation itself that I am different from my parents, doesn’t really affect me that much. […] It’s more that my younger brother is very much the same. That creates a bit of a “them and me” contrast in my head. A bit like it’s the front of the family and I’m the one who falls outside of that. (NL3, Dutch, 22 years, transgender male, recurrent)

[…] there is my family that has never understood me. Again, you know how it is in Indian families, they don’t particularly talk about things, right! And I am not a person who cries, but in the past whenever I have cried due to something that was really concerning or something that was heavy for my system and I wanted to relieve it, all of this has somehow never been received well. I have been told things that how I am exaggerating or ‘drame kar rahi hai’ (=she is doing drama / being a drama queen). It made me question whether am I feeling things to an extreme? Or is it like justified that people are not understanding? (D2, Indian, 22 years, female, recurrent)

Notably, while a large group of participants with recurrent experiences of not fitting in reported having felt different from family, hardly any participants with situational experiences did. Participants with situational experiences of not fitting in also reported feeling different from family in concrete situations only, while those with recurrent experiences rather reported differences that kept coming back across different situations (e.g., in personality or values). A similar pattern emerged for feeling different from classmates at school, which was discussed much more by participants with recurrent than situational experiences of not fitting in.

There are multiple interpretations of these findings. For one, it is possible that some difference from societal norms showed early on, for instance in the family context or – more often – when children entered school. Similarly, some people may have a higher sensitivity to rejection or difference by disposition, making them more likely to perceive differences throughout life. However, in line with theorizing about the development of *rejection sensitivity* [70], participants often explained that previous feelings of deviation from others had made them more alert to exclusion, doubting their relationships more, or having less trust in their relationships.

[If you have different interests than others at school] you’re uncool right away or at least I was afraid that they see me either as a nerd or uncool, although I was not. […] I did feel a lot of anxiety. […] Anxiety of judgement of other people towards me. So, with myself, I felt fine. And that goes on until today, but I’m afraid of judgement of others towards me. […] So, also in relationships, not being able to trust anybody. Um, that is- that is a huge- a huge thing. […] And then, of course, the feeling persisted to a certain extent, and, let’s say, the timidness, the anxiety a little, to not fit in or being weird […]. (PS7, German, 28 years, female, recurrent)

### Differences in perceived norm deviations and causes between cultural samples

As discussed for group differences between people with situational and recurrent experiences of not fitting in, qualitative data are not suitable for conclusions about frequencies in different groups. Accordingly, we discuss only qualitative or striking quantitative differences, which may serve as basis for future research (for additional quotes, see Table S2.3).

Differences in findings across samples reflected possible cultural differences in traditionality regarding partnership, sexuality, and religion. For instance, while participants across samples reported pressure to be in romantic relationships, only Chinese and Indian participants reported societal pressure to get married by a certain age.

Basically, typical Indian family issue of marriage. Enforcing arranged marriage on me. I want a good partner who respects my ideology and has their ideology. Otherwise, I don’t want to get married. I have other dreams that I want to pursue. But they are like ‘no! you will have to get married. At this age, you should be settled’. So, this emotional trauma, arguments, discussions goes on… (D4, Indian, 30 years, male, recurrent)

These reports align with descriptions of societal pressures to get married by a certain age in India and China [7, 33], stigmatization of particularly women in China who are not married by the end of their 20s, and trends of young Chinese people to postpone marriage [33].

In most samples, a small minority of participants mentioned feeling different for having a non-normative sexual orientation or not fitting in with traditional gender norms. By contrast, more than half of the Dutch sample mentioned these themes, versus none of the participants in the Nigerian sample. People may be more likely to engage with sexual orientation and gender norms if there is high societal acceptance of being LGBTQ + . While this seems to be the case in the Netherlands, there is low acceptance in Nigeria, and relatively low acceptance in China and Turkey ([6]; see also Table S1). Notably, the Chinese and Turkish samples included mainly highly educated people living in larger cities, where social acceptance of being LGBTQ + may be higher as well.

In comparison to participants from other samples, Nigerian participants talked particularly often about not fitting in with peers because of adhering more closely to their Christian faith or being less religious than their families. Indeed, in comparison to other parts of the world, personal importance of religion seems to be very high in Nigeria (88% of Nigerian survey respondents reported religion to be very important in their lives; [59]), making religiosity a relatively relevant topic of comparison.

I’m always serious with my church program. I don’t joke with my church. They [friends] even always make jest of me for that. […] Well, because starting from them, whenever they want to go out, they don’t even bother telling me again. And they used to say to me that I’m different from them. They sometimes also like to follow after guys. They just removed me from their things, because they know that I won’t do whatever they want to do. That’s because mine is all about godly things. (NG6, Nigerian, 19 years, female, situational)

Daddy is an evangelist. And me, I am not a religious type. But others in my family are this over religious persons; everything is church, church, church. […] And I was just trying to bring this perception that, “it is not everything we do that should be about church, church, church”; there should be some things that we apply common sense to. But, I was made to know that in this family, it is either God, church, or nothing. (NG5, Nigerian, 24 years, male, recurrent)

Mainly participants from Turkey, China or Hong Kong, and India reported intergenerational differences in traditionality, leading to feelings of not fitting in when with members of other generations.

I think it’s about the past and the way people used to think back then. The people of that time had more rigid thoughts. For example, my grandmother’s thoughts are even more rigid, my mother has opened up a bit more, and so on. These rigid ideas have become such fundamental truths, and going against those truths or not respecting their thoughts separates me from them in a very stark way, and I become the "bad one." (T2, Turkish, 26 years, female, recurrent)

With the hippie movement in the 1960s and 1970s, values and attitudes --- for instance, towards sexuality and marriage --- became less traditional in many western countries. Accordingly, young adults in western contexts may often perceive less difference from their parents’ generation than young adults in more traditional societies. Additionally, many participants in the Turkish and international samples seemed to hold relatively liberal values, resulting in larger perceived intergenerational gaps. Indeed, more western and individualistic values seem to have emerged in cultures with more family-oriented and collectivistic values over the past decades (e.g., [12, 16]).

In line with the notion that Confucian cultures place a strong emphasis on achievement as a means of fulfilling family obligations (Qu, 2023), a participant from southern China reported a stronger focus on achievement in Chinese culture than participants from other cultures.

In China, they have a rank of the- the university. So, I’m definitely not the top ten rank of the university. But they use that to compare to the others. […] Or some of the parents even brag for us or, like, say, ‘Oh, my kids is from this university and my kids can be the top one, but how about your kids?’ […] So, a lot of parents are quite nervous about this. And - so, they’re super, super crazy about it and some of the parents, which was [who were] super focused on children’s educations, they would choose where they live. [i.e, in neighbourhoods with particularly good schools] […] And living in this environment, if you [laughs] got a low academic performance, you are going to be labeled a bad kid. (HK13, Chinese, 29 years, female, recurrent but not throughout life)

While participants across cultures mentioned different treatment by others as the reason why they perceived not to fit in, this was particularly common among Nigerian participants. A philosophy or worldview that is described as widespread in many parts of Africa, including Nigeria, is *ubuntu* – ‘being self through others’, also often defined as ‘I am because we are’ (*Ubuntu ngumuntu ngabantu*; [20], p. 344). Relatedly, cross-cultural research suggests that in more collectivist cultures, people construe their selves more strongly in relation to others [54]. Accordingly, it is conceivable that in the Nigerian context, the reactions of others convey more information about being different than in cultures with more independent self-construals.

Furthermore, only in the Nigerian sample, a participant with recurrent feelings of not fitting attributed her feelings of being different to possibly being cursed.

I feel like I am cursed, and I don’t think I have ever told anybody this; no. Most time, when I sit and think about this, I feel like maybe I’m cursed or something. (NG5, Nigerian, 24 years, male, recurrent)

While cultural beliefs about mental health conditions being caused by spiritual curses exist across the globe, this is well-documented in the Nigerian context [42], and Sub-Saharan Africa more generally [66].

Being less outgoing, less talkative, more introverted or shyer than others were mentioned as reasons for feeling different in all samples, but this was much more common among participants from India, China and Hong Kong, and Nigeria than in other samples. By contrast, earlier theorizing assumes that self-effacement (e.g., shyness; not expressing emotions and thoughts much) is valued in more collectivistic or group-oriented contexts because it can preserve group harmony [12, 64]. While support for this theorizing has been found in East Asian contexts (e.g., Japan, China; e.g., [12]), collectivistic cultures differ. Indeed, highly collectivistic Arabic cultures have been found to value assertiveness, which is one form of self-*enhancement* [64]. In Indian or Nigerian contexts, greater talkativeness and sociability may be valued as they promote the culturally normative energetic and active engagement in groups rather than disrupting harmony within the group. Furthermore, values in many collectivistic cultures, including Chinese culture, seem to have become more focused on assertiveness and individual achievements over the last decades --- particularly in cities, where interviews in the current study were conducted [12].

Finally, although the international sample from the pilot study does not allow to explore norm deviations in specific geographical contexts, it allows to study norm deviations among highly geographically mobile young adults: Most participants in this sample had moved countries at some point. Accordingly, having a different cultural background or mother tongue than others were discussed by most participants in this sample as reasons for not fitting in, highlighting the potential relevance of not fitting in with others when people migrate.

### Perceived consequences

Figures 3 and 4 illustrate reported consequences of not fitting in, which seem in line with previous empirical findings on intrapersonal consequences and interpersonal reactions to people who ‘violate’ social norms (e.g., [69], to minorities or stigmatized groups [19], and to employees who do not fit their organization [72]. They are also consistent with theorizing on consequences of not fitting in with social norms [37]. Notably, almost all consequences were reported both by participants with and without minority status. Some concrete consequences (e.g., self-harm; perceiving interactions as tiring or effortful) were mentioned only by individual participants from either group, but they are unlikely to be exclusive to one of the two groups. No consequences were reported by multiple minority participants, yet not at all by non-minority participants.

Despite convergence with existing literature, participants’ own reports also highlight that consequences are more diverse and wider-reaching than what previous studies may suggest (see Table S2.4 for quotes). Notably, while most participants reported mainly negative consequences of not fitting in, some participants also reported positive consequences. These included being met with more trust, openness, appreciation, or respect than others; feeling proud of one’s uniqueness; more freedom and less stress; or better understanding of what one wants oneself. These aspects may be useful to consider in strengths-based interventions.

More negative consequences frequently reported by both people with situational and recurrent experiences of not fitting in included emotional discomfort, frustration and anger, not being understood by others, loneliness, rejection and exclusion, or being influenced in personal choices.

I had to drop something that I really like [playing basketball], um, because of social pressure [not to play basketball as a girl] or wanting to belong with whatever norms that were in the school. I honestly think that I could have been a really good basketball player. Like, I could have been professional in it. (PS2, Venezuelan, 25 years, female, situational)

Indeed, multiple participants talked about leaving jobs, leaving educational settings (e.g., going from so-called ‘honors’ programs into regular education), or hobbies because they felt that they did not fit in. Perceptions not to fit norms may thus be possibly relevant influences on people’s life paths.

By contrast, participants with recurrent experiences of not fitting in seemed to report more sadness, anxiety, stress, confusion, and rumination about not fitting in than participants with situational experiences.

So, sometimes, I feel like, is it that I frown my face, or I don’t have a good aura about myself? I don’t know. I just wonder. […] I just feel someone should tell me. Not knowing something is like the worst feeling ever. If I know what is happening, at least I will know what to do. (NG3, Nigerian, 26 years, female, recurrent)

Most striking differences in consequences reported by participants with situational versus with recurrent experiences occurred in relation to interpersonal relationships or reactions by others. For one, participants with recurrent experiences of not fitting in more commonly reported being told, explicitly or implicitly, that they were different or ‘weird’.

And sometimes they’d make comments like - “you’re so different” or “your thought process is strange”. They’d say it jokingly, but it stung. (D8, Indian, 25 years, female, recurrent)

Like, “if we are saying ‘A’, you will be saying ‘Z’, it is abnormal”. In fact, some people even see me as a weirdo. (NG5, Nigerian, 24 years, male, recurrent)

Related to the quotes above, participants with recurrent experiences more commonly reported being treated as inferior, and being met with disapproval or devaluation because of being perceived as different, than participants with situational experiences. They also more often reported to be perceived in – according to them – inaccurate ways (e.g., as complicated or arrogant). Accordingly, a similar pattern emerged for feelings of inferiority, insecurity, or self-monitoring.

Feeling worthless as well. […] with exclusion, there is a sense of being underestimated and feeling small. There is a sense of inadequacy. (T9, Turkish, 27 years, female, recurrent but not throughout life)

[Being excluded in primary school] created a very strong feeling that there was something wrong with me, but I didn’t know what. […] [It resulted in] A lot of insecurity and shame […] Um, yes, but also a lot of, um, insecurity and shyness. Um, in elementary school I had a really hard time with—still do—my voice was very soft and I heard “can you speak louder?” every 5 seconds. Um, and I just couldn’t do that very well. And now, I notice that occasionally, when I’m in a new situation that I’m nervous about, it happens again. (NL6, Dutch, 22 years, female, recurrent)

Moreover, participants with recurrent experiences of not fitting in more often reported to experience conflict or instability in relationships as consequences of not fitting in, not being able to participate in group conversations or other activities (see first quote below), or difficulties finding or retaining relationships (see second quote below).

And I remember my mom telling me ‘Well, <name>, if you don’t know what to say in a group, you can just smile and that’ll always work’. So, I would smile a lot. […] Because I, I didn’t really know what to say. (PS12, US American/Belgian, 29 years, female, recurrent)

This feeling of difference has not made me keep any friend for up to two years. The longest I have had a new friend is like a year plus. Just a year plus; and after a year, it is gone. (NG5, Nigerian, 24 years, male, recurrent)

While multiple participants in each category reported experiences of exclusion or rejection due to not fitting in, only participants with recurrent experiences of not fitting in reported bullying, or other instances of aggression (e.g., being shouted at).

Against this backdrop, it is not surprising that, while both participants with situational and with recurrent experiences reported feelings of non-belonging, only participants with recurrent experiences reported to generally feel a lack of belonging in their lives.

The strongest thought was that “I’m weird. There’s something wrong with me”. And that thought kept repeating - in school, in college, in family gatherings. Sometimes I felt like I didn’t belong anywhere. (D8, Indian, 25 years, female, recurrent)

### Coping strategies and suggestions for interventions

Reported coping strategies are summarized in Fig. 5. The most common ways of dealing with not fitting in were to leave the social context in which one did not fit in, and find or connect with similar, accepting, or understanding others or communities. Particularly members of minority groups (mainly sexual minorities in our sample) reported connecting to others *with similar experiences*, which may have been more difficult for people who did not belong to any defined minority group. While leaving the context of not fitting in was usually described as effective coping strategy, it might sometimes also have signaled rejection sensitivity, as in the example below by a participant with recurrent experiences:

At the campsite, there were other friends of mine who weren’t part of that organizing committee, and they felt it didn’t matter. […] My friend said if I didn’t care so much about it, I could have smoothly integrated with the group, like just walking a few steps faster to catch up and chat with them. But once I feel that emotion [not being part, being left out], I don’t want to join the group at all, I want to distance myself from them, feeling very lonely and miserable. When others don’t notice me, I feel like lashing out and leaving. (HK1, Hong Konger, 19 years, female, recurrent)

Similar to qualitative findings on how people cope with loneliness (i.e., a related negative social experience; [38]), participants often reported coping strategies such as accepting, supporting and relying on themselves more (see first two quotes below); or accepting not fitting in and, relatedly, becoming less sensitive to it (see last quote below).

I’m also trying to self-“securify” myself […] - to tell my kids of- the kids versions of mine, ‘This is not your fault.’ (HK13, Chinese, 29 years, female, recurrent)

Ah, I have learnt something over the years, and what I have learnt is that, “if nobody is accepting who I am, then I will accept myself”. So, I accepted myself for who I am. (NG5, Nigerian, 24 years, male, recurrent)

That’s why it feels like it’s a difference that you accept, and at some point it’s a difference that makes me feel good rather than bad. […] In fact, I embraced this "weird" label. I understood it as a form of admiration in their language. I didn’t take it as bullying. (T1, Turkish, 28 years, male, recurrent)

Oher participants reported coping with feeling different through realizing that experiences of not fitting in often pass with time; or distracting themselves (e.g., through enjoyable activities; consuming movies or social media). Particularly participants with recurrent experiences of not fitting in also reported the usefulness of talking about their experiences of not fitting with others.

Coping strategies are not always satisfactory or helpful. For instance, multiple participants with recurrent experiences reported dealing with the fear of rejection or devaluation by hiding parts of themselves, including disclosing only limited information about themselves, or keeping their conversations or responses at a superficial level --- as compared to hardly any participants with a situational experience.

For a long time, I’d censor myself just to impress others. It felt like I was performing, not living. (D8, Indian, 25 years, female, recurrent)

Even now at university, I try to be neutral with everybody. […] So, I really shut down and basically tried to avoid conversations or if I had a conversation, very superficial and neutral. (PS7, German, 28 years, female, recurrent)

This is in line with literature on ‘masking’ in neurodivergent minorities (e.g., [61]) or ‘being in the closet’ in sexual minorities (for quotes see Table S2.5). However, it was also reported by participants who did not belong to defined minority groups, as illustrated by the quotes above. Several participants also reported internally disengaging from conversations or becoming uninvolved in them.

Sometimes when I’m in a friend group, I feel uncomfortable because I can’t communicate, and I get bored. Then I just look around and wait for time to pass. (T6, Turkish, 26 years, female, recurrent)

Relatedly, multiple participants with recurrent experiences of not fitting in reported social withdrawal in reaction to not fitting in, sometimes driven by rejection sensitivity.

These days, I no longer converse with others. I just engage my books, watch movies, play games. […] Situations that might have allowed me to meet new people or make more friends is forfeited, because of the fear of feeling differently. Even when someone tries to initiate friendship or conversation with me, I will just cut off from the person, because at the end of the day, the person might still leave or ignore me. (NG3, Nigerian, 26 years, female, recurrent)

I avoid people. And I feel that when I’m around people, I always end up feeling like the odd one out. So, I used to try being around people, but now I avoid it. […] Frankly speaking, for the last 8 months, I haven’t really been in anyone’s company. […] So, for your own safety, stay away from people. That’s the best decision I’ve made […]. (D5, Indian, 27 years, male, recurrent)

While social withdrawal was often presented as an unsatisfactory way of dealing with not fitting in among participants with recurrent experiences, participants with situational experiences mainly reported social withdrawal as a useful coping strategy.

I really had the need to be alone a lot to, like, spend time, and […] reconnect with myself to not get lost in - in all this, like, readjusting and trying to fit in. […] I feel like it was a really, like, strengthening experience, being who I am. […] Cause for me, it helped that I was spending time alone, like, paradoxically. (PS5, Polish, 29 years, female, situational)

Similarly, while many participants with recurrent experiences of not fitting in reported that their attempts to adapt or conform to others were unsuccessful or unsatisfactory, voluntarily adapting to one’s social context was sometimes perceived as useful. Participants with only few situational experiences of not fitting in explained that it was exactly their ability to easily adapt that had contributed to them hardly ever feeling different.

Yes, I can fit in with any group I encounter […] Just talk about the things people want to talk about. […] If they’re not chatty, just keep talking about yourself or keep asking them questions. Eventually, they might open up and share something. But if you meet someone who’s really outgoing and cheerful, you could chat about where to find good food, what clothes they like, or all sorts of random things – that sort of conversation flows easily. (HK12, Chinese, 26 years, female, no experiences of feeling different)

Actually, that [not fitting in] doesn’t bother me. It’s more intriguing for me. It turns into something like a challenge or a competition for me to fit in with that group. (T3, Turkish, 26 years, male, situational)

Multiple participants – mainly those from the international sample, India, and the Netherlands—also offered suggestions for interventions. These included support groups or increasing tolerance and acceptance of diversity – in society more generally, but particularly in primary or secondary schools, where many participants reported to have experienced not fitting in.

Raise awareness among students the same age, um, that there might be people in your class that are a little weird or feel different, but instead of acting in common sense: ‘Well, you’re weird, okay, bye.’ Um, rather be more inclusive. […] And talk about ‘Maybe you yourself felt different in certain situation.’ Because, I think, everybody feels different. (PS7, German, 28 years, female, recurrent)

Particularly participants with recurrent experiences also mentioned the relevance of professional help (e.g., psychotherapy or counseling), which may --- among other things --- help reduce negative thought patterns of rejection sensitivity, as illustrated by the quote below.

I’ve been in therapy for quite some time, so I’ve started to find myself less strange, but I just know that I have different experiences than the average person. But that’s not a bad thing, because it has improved my self-image, making it easier for me to approach people and not always have the standard thought in my head: People are going to think I’m weird, so they don’t like me. (NL3, Dutch, 22 years, transgender male, recurrent)

### Culture-specific consequences and coping strategies

In line with religion being, on average, highly important to Nigerians (see above), multiple Nigerian participants mentioned turning to religion or faith in God to deal with the negative feelings of not fitting in with others. Possibly relatedly, Nigerian participants often talked about controlling negative thoughts and instead attempting to view things positively, which was only rarely reported by participants in other samples. A few participants in other contexts also mentioned dealing with their feelings of difference through spirituality. For instance, one Chinese participant reported that giving up his ‘self’, adapting to outside circumstances and being at service to other people had helped him feel less different and lonely.

I don’t have a sense of self anymore, can I say that? […] differences only exist between two things. A and B are different, but if you don’t have B, then it doesn’t matter. So, now when I look at others, hmm, what I see is just others. I no longer feel the differences between others and myself. […] For example, in the Dao De Jing [a Daoist text], there’s the concept of water, right. You know, water is fluid, it changes, it’s that kind of feeling. You observe and understand what they [another person] like to talk about. Uh... when you talk to them about these hobbies, they feel very happy. […] Now, the feeling of connection is no longer important to me. Because to have a sense of connection, you need to have a self, and then you need to connect with others. But now, most of the time, I don’t feel myself. (HK2, Chinese, 19 years, male, recurrent)

The idea of giving up on the self or ‘ego’ entails letting go of attachment to fixed identities, material possessions, or control. It is a key principle in many religions and spiritual teachings (see also a quote by a Venezuelan participant in Table S2.5), including Taoist, Buddhist, and Confucianist traditions. These are practiced and have shaped thinking in China. Relatedly, it seemed that particularly participants in the Chinese / Hong Kong sample perceived adapting to other people as relevant way of socially integrating, which was often perceived more negatively by participants in other samples (see above and Table S2.5).

While participants in different samples talked about disappointing others, only a participant from South China mentioned bringing shame to her family.

But, like, when they’re like have a parent teaching day, they [teachers] will just […] shame your parents and say, ‘Look, your kids are the- like, the not-good-enough.’ […] [Other parents will tell them:] ‘Oh, what does [daughter] study? Oh, really? Don’t you want to, like, let her to go abroad for study or something?’ But they’ll say, ‘Yeah, I try, but she doesn’t want.’ – ‘Oh, but try harder. […]’ So, somebody think they have the right to teach my parents how to do. […] Come on, like, why you shame my parents just because- like, let’s say, if this is truly my fault, why you do that? (HK13, Chinese, 29 years, female, recurrent but not throughout life)

This is in line with findings of a strong focus on achievement, importance of strict parenting, and interdependence in families in many Asian cultures [11]. Commenting on *others’* parenting may furthermore be related to the notion that people in certain collectivistic cultures impinge their own interests on other ingroup members more than in more individualistic cultures [49]. The ingroup is often conceptualized as the family, but people’s narratives in our qualitative interviews suggest generally strong involvement with other people’s behavior and choices in more collectivistic cultures. Some authors have also suggested that social control of delinquent behavior is relatively common in East Asian countries, such as China (e.g., Jiang et al., 2010).

Finally, it was also striking that most Nigerian participants reported engaging with their phones to deal with unpleasant feelings of not fitting in (e.g., social media, watching videos), as compared to only few participants in all other samples. Indeed, data on internet usage worldwide suggests that Nigeria is one of the world’s countries with most video calling, most frequent social media use, watching online videos, listening to music online, listening to podcasts, accessing news online, online purchases, or checking health symptoms online [43]. As such, it may be unsurprising that phones are also particularly often used to distract oneself from negative feelings of not fitting in.

## Implications

The current qualitative study provides an overview of the broad range of norm deviations people perceive across different cultures. By filling existing theories and research (e.g., [22, 37]) with lived experiences from different cultural contexts, this research thus promotes our understanding of what not conforming to social norms means in practice. Commonly reported norm deviations included – among other things - having different preferences and perspectives than others; different achievements or skills (e.g., being highly intelligent; being less skilled or knowledgeable than others); or having a different appearance (e.g., dressing differently; being taller or bigger). Two frequently reported concrete norm deviations were being less outgoing, talkative, extraverted, or sociable than others or having different developmental timing (e.g., having a first partnership/romantic relationship later than others; feeling more mature than others). Rather than reporting deviations for *personal *characteristics only, participants also reported feeling different for having different relationships than others (e.g., having stricter parents, being less acquainted with others, or feeling treated differently by others). 

Participants generally reported quite similar types of norm deviations across cultural samples. Nevertheless, we also observed a few differences. In line with the idea that people experience norm deviations particularly in relation to characteristics that are culturally relevant or valued (e.g., [37]), participants reported deviations from norms to get married (by a certain age) particularly in the Chinese or Indian samples; norms regarding religiosity particularly in the Nigerian sample; norms to fare academically well particularly in the Chinese/Hong Kong sample; or norms to be outgoing or sociable particularly in the Indian, Nigerian, or Chinese/Hong Kong samples. Participants also reported norm deviations particularly if these norms were currently societally questioned – such as the norm to be heterosexual, deviations from which were discussed more in the Dutch than other cultural samples.

Recurrent experiences of not fitting in with social norms have, thus far, mainly been studied for defined minority groups (e.g., sexual or ethnic minorities). However, in the current study, most participants with recurrent experiences of not fitting in were not part of a minority group. Instead, they reported not fitting in with their culture or society because of generally different interests, opinions or values (e.g., regarding level of religiosity), different life experiences (e.g., having lost or cared for a family member), or being less sociable or outgoing than others. Multiple participants reported recurrently experiencing not fitting in because they felt treated differently by others without knowing what exactly made them different. Unlike participants with situational experiences, they had often experienced not fitting in with their families or in primary school already, raising the question if these participants’ difference showed already in their childhood or if early experiences of being different paved the way for later perceptions of being different from others (e.g., through rejection sensitivity; [70]). Such recurrent experiences of not fitting in without being part of a defined minority may require more attention in research and intervention – particularly given the various negative consequences of norm deviations for social and emotional well-being reported in this research.

More specifically, such reported consequences included – among other things—feedback by others that one was different (e.g., being told to be weird; being called names), rejection and exclusion, being met with disapproval or devaluation, negative emotions (e.g., feeling uncomfortable, lonely, angry, sad), feeling inferior, being influenced in personal choices (e.g., moving to a different place), or hampered interactions (e.g., not feeling understood, difficulty finding relationships). Participants with recurrent experiences seemed to discuss certain consequences more than participants with situational experiences of not fitting in. For instance, they reported having been told more that they were different, having been met more with disapproval or devaluation, and, accordingly, to experience feelings of inferiority more. However, similar consequences were reported across different cultures and by both minority and non–minority participants, again highlighting the need to address negative consequences of not fitting in also among non-minority individuals. Indeed, although people who are not part of a defined minority may often face less stigma than members of minority groups, they often lack a relevant resource that members of defined minorities can have; a group to identify with or gain support from.

Current interventions to improve social wellbeing strongly focus on reducing loneliness and social isolation, often through initiatives to increase social contact. While this may serve needs of people who lack social opportunities (e.g., older adults), our findings align with previous research suggesting that such approaches alone are not sufficient. In this and previous research [36, 37], it appears that loneliness and, sometimes, social isolation can result from not fitting in with others—among other things, through hampered reciprocal understanding, repeated rejection or exclusion, devaluation by others, and sometimes social withdrawal. These findings suggest that social connection often requires more than mere opportunity to interact.

This is particularly important because, based on our findings and the existing literature, one may speculate that interventions that merely promote social contact may even trigger loneliness and withdrawal. People who recurrently felt that they did not fit in with others often reported consequences such as (perceived) rejection, devaluation, and lack of understanding across various social situations. It is therefore likely that they also experience such uncomfortable consequences when brought into contact with random other participants in an intervention. This is especially likely because people who have been or felt rejected before can become more sensitive to possible rejection [70]. Additionally, interventions promoting social contact may reinforce the reason why participants who described themselves as introverted reported not to fit social norms: they felt pressured to conform to a norm of being outgoing and talkative.

Participants’ reports suggest that interventions to counteract negative consequences of not fitting in with social norms may need to target both individuals who experience feeling different and their social contexts: For one, participants stressed the relevance of professional help: Indeed, psychotherapy may help process experiences of not fitting in made earlier in life, and counteract thought patterns of rejection sensitivity (e.g., [23]). Participants’ suggestion of support groups align with their reports that sharing one’s experiences with others could ease negative feelings of not fitting in. Different from interventions promoting social contact without matching participants on certain characteristics, support groups can target people with a concrete shared characteristic (e.g., sexual orientation; introversion), or people who share the experience of feeling different from others more generally. Several participants also suggested to implement programs to foster tolerance and acceptance of diverse characteristics (e.g., [1]), particularly in primary and secondary schools, where participants had often experienced not fitting in particularly strongly. While interventions tend to focus on fostering tolerance towards members of concretely defined groups (e.g., ethnic or religious groups, [44]), our findings suggest that it would also be relevant to address more subtle differences, such as in interests, appearance, or developmental timing. Finally, observations of cross-cultural differences in our data also suggest the possible usefulness of considering culture-specific ways to deal with experiences of not fitting in when developing interventions. Religion, for instance, was often described as a source of support in the Nigerian sample, while mobile phones appeared to be a possibly useful means of reaching people in this specific context. Future research will need to test these preliminary suggestions for interventions before they can potentially be put into practice.

## Limitations and future research

Given the large proportion of participants with recurrent experiences of not fitting in, this research provides insight into experiences of people who might need intervention. However, it also underscores the likelihood of self-selection bias, given that the current sample was recruited through researchers’ social networks or social media, openly disclosing the theme of the study. Relatedly, despite the aim of reaching samples diverse in education background, most samples --- except for the Nigerian sample --- contained a relatively large share of participants with university education. Accordingly, the current research is not suited for drawing conclusions about prevalences (e.g., across different cultural groups), which would require quantitative research with representative or random samples. However, current findings can provide a basis for such quantitative research by suggesting which aspects may be relevant to include or which phrasing or examples to use when developing questionnaires (see quotes in Table S2 in the supplemental materials). The latter is relevant because commonly used abstract questionnaire items can be difficult to understand for lay people, especially if they have not pursued higher education [45].

While the aim of this research was to provide an overview of different consequences that not fitting in with social norms can have, consequences are likely to vary depending on the concrete type of norm deviation and the social / cultural acceptance thereof in a specific context. For instance, differences from others that are more clearly visible (e.g., being part of an ethnic minority; having a visible physical disability; being much taller than others) are likely to elicit more reactions by others (e.g., gaining unwanted attention; being called names) than norm deviations that can be hidden (e.g., sexual orientation; different interests or preferences than others). Being part of a sexual minority may more often elicit negative reactions by family members than being part of an ethnic minority, yet less so in environments where being LGBTQ + is more socially accepted (e.g., in the Netherlands as compared to in Nigeria; [6]; in urban versus rural contexts; e.g., [52]). Future research to develop interventions may study negative consequences and, accordingly, possible strategies to reduce them, for specific norm deviations in specific contexts.

Qualitative research allows to examine *perceptions* as opposed to actual deviations from social norms. While perceptions of norm deviations can certainly have negative intrapersonal consequences (e.g., feeling lonely; insecurity), there likely are more *inter*personal consequences (e.g., rejection and exclusion; being treated as inferior) for norm deviations that others perceive as well. As such, future research may disentangle antecedents and consequences of norm deviations perceived by oneself versus others. This may help inform the focus of an intervention for concrete norm deviations (i.e., individual versus context). Relatedly, future research could examine the extent to which people erroneously perceive to deviate because of erroneous perceptions of social norms (*pluralistic ignorance*, see Introduction). In such cases, the most straightforward solution to feelings of not fitting in would be to provide psychoeducation to correct erroneous perceptions of social norms.

## Conclusion

This research highlights the broad range of experiences of not fitting in across different cultures, and their relevance for social and mental wellbeing. While most people experience not fitting in with others occasionally, it seems particularly relevant for practice to study experiences that persistently occur across social contexts and throughout a large share of a person’s life. Support groups, programs fostering tolerance to and acceptance of diversity, and psychotherapy may be approaches to target feelings of not fitting in, if confirmed in future empirical research. This may be particularly important for people who are not part of minority groups because their experiences are hardly discussed, while they may more often lack a social group to identify with than members of minority groups.

## Supplementary Information

Supplementary Material 1.

## Data Availability

The interview guide and codebook for this study can be found on the Open Science Framework (osf.io/mz4wk). To preserve participants’ anonymity, transcripts and the categorization of each case as situational versus recurrent loneliness are not publicly available. Information can be requested from the corresponding author. This study was not pre-registered.
