# Supplementary material for: What makes you different? A qualitative study on experiences of not fitting in with social norms across cultures
Source: BMC Psychol. 2026 Jul 11;14:1530. doi: 10.1186/s40359-026-05130-z (PMC13689083; doi:10.1186/s40359-026-05130-z)
Supplement: Supplementary file 1 [file 40359_2026_5130_MOESM1_ESM.docx]

**Supplemental Materials**

| Table S1: Cultural Characteristics in Different Study Contexts | p. 2 |
| --- | --- |
| Table S2.1: Additional Quotes for Types of Social Norms that Participants Perceived to Deviate From | p. 4 |
| Table S2.2: Additional Quotes by Participants with Recurrent Experiences of not Fitting in | p. 14 |
| Table S2.3: Additional Quotes for Differences in Perceived Norm Deviations and Causes Between Cultural Samples | p. 21 |
| Table S2.4: Additional Quotes for Perceived Consequences of Not Fitting in With Social Norms | p. 24 |
| Table S2.5: Additional Quotes for Coping Strategies and Suggestions for Interventions | p. 31 |
|  |  |

**Table S1**

*Cultural Characteristics in Different Study Contexts*

|  | Min; Max in study | Nigeria | India | Turkey | China / Hong Kong | The Netherlands |
| --- | --- | --- | --- | --- | --- | --- |
| **Tightness - looseness^1^** |  |  |  |  |  |  |
| Eriksson et al., 2021 | 1.46; 2.93 | 2.26 | 2.48 | 2.17 | 2.05 / - | 1.59 |
| Gelfand et al., 2011 | 1.6; 12.3 | - | 11 | 9.2 | - / 6.3 | 3.3 |
| **WVS** |  |  |  |  |  |  |
| Traditional family values index^2^  (Wave 7, 2017-2022) | .20; .69 | .60 (.10) | - | .55 (.13) | 54 (.12) / .40 (.11) | .20 (.13) |
| Only items included that are also available in Wave 6 | .18; .64 | .55 (.11) | - | .51 (.15) | 43 (.16) / .31 (.13) | .18 (.14) |
| Traditional family values index (Wave 6, 2010-2014) | .29; .68 | .61 (.10) | .68 (.09) | - | 49 (.12) / .47(.12) | - |
| Traditional attitudes towards homosexuality^3^ (Wave 7, 2017-2022) | .11; .84 | .65 (.11) | - | .58 (.16) | .55 (.17) / .31 (.17) | .11 (.14) |
| Only items included that are also available in Wave 6 | .05; .84 | .65 (.11) | - | .59 (.17) | .57 (.20) / .30 (.20) | .05 (.13) |
| Traditional attitudes towards homosexuality (Wave 6, 2010-2014) | .08; .68 | .58 (.15) | .54 (.22) | - | .47 (.22) / .37 (.20) | - |
| Traditional attitudes regarding gender roles^4^ (Wave 7, 2017-2022) | .13; .57 | .46 (.15) | - | .39 (.16) | .34 (.13) / .32 (.14) | .13 (.13) |
| Traditional attitudes regarding gender roles (Wave 6, 2010-2014) | .12; .50 | .45 (.18) | .40 (.18) | - | .33 (.16) / .31 (.15) | - |

*Note.* ^1^Higher values indicate higher tightness. ^2^In the index to assess traditional family values, we summarized participants’ self-reported importance of family, perceptions of duty towards parents (i.e., to make them proud or take care of them), duty towards society to have children, and attitudes towards being unmarried, towards sex before marriage and towards casual sex. Wave 6 of WVS data does not contain certain items that were included in indices for Wave 7 (“People have the duty towards society to have children”, “It is children’s duty to take care of their ill parents”, “Having casual sex is justifiable”). This is why an additional indicator was created including only items that are also included in Wave 6 of WVS data. ^3^In the index to assess traditional attitudes towards homosexuality, we summarized items assessing if homosexuality was perceived as justifiable, preferences for not having homosexuals as neighbors, and the opinion that homosexual couples are as good parents as heterosexual couples. Wave 6 of WVS data did not contain “Homosexual couples are as good parents as other couples”. ^4^In the index to assess traditional attitudes regarding gender roles, participants reported if they agreed that men make better political leaders or business executives than women, that they should have more right to a job and to go to university than women, and that it is problematic if women earn more than men.

**Table S2.1**

*Additional Quotes for Types of Social Norms that Participants Perceived to Deviate From*

| **Social norm related to …** | **Participant quotes** |
| --- | --- |
| Living with disability or illness | But from the age of 4-5 years, I understood that yes, I am somewhat different. I am different. And I am not the same as the people around me. The children who are playing with me, they are stronger. They are more physically fit. I am not like that. […] It usually happens when everyone my age is able to do something, and I can’t—because of one reason or another. For example, if everyone my age can drive—like a two-wheeler—and I can’t do that because of my disability. […] That’s when I realize I’m doing things differently, and I am different. […] And that’s the thing—where there is a difference, there is loneliness. […] Let me share a small incident that reflects this—something I experience almost every day. Let’s say there’s a warm-up party at my office at <company name>. I have the best clothes to wear, the best accessories—things I’ve bought myself with my own savings. When I get ready for the party, I feel good. I feel like I’m standing there with my head held high. But still, somewhere inside, I think: If I were physically fit, my personality would come through even more. I’d stand out in the crowd—like I was the star of the evening. It would feel like people are noticing me. […] I believe that if I weren’t disabled, that energy, that impact, would come out naturally. And if I’m being completely honest—I don’t want this disability in my next life. Even if I don’t get happiness, even if I don’t get a family, that’s okay. But I just don’t want to be born with a disability again. (D5, Indian, 27 years, male, recurrent)  I used to be grouching, just not really excited about things. Or the complete opposite, where I would be very manic, and I would love to buy things online, or I would watch a whole TV show in an entire day. But again, felt so alone with that, and felt like it was just me that was going through that [not understandable: could be ‘bull’] again. (PS8, British, 22 years, male, recurrent)  And I shared this [a situation that made participant agitated] with my friends, hoping to get some understanding or at least confirmation. But all I heard from everyone was, “Okay, you're being dramatic. Calm down, it's not that big of a deal.” And then I thought, “Oh guys, you're supposed to be my emotional safety net.” That's a shame. But then again, if you don't know how a mental disorder can affect your thought patterns and how that can make me feel emotionally, then I understand. (NL3, Dutch, 22 years, transgender male, recurrent) |
| Cultural background or ethnicity | Yes, I've felt that quite often. I think it's partly because my mother is Chinese Indonesian. So, you can see that in me too, in terms of my appearance, that I don't look like a typical Dutch person. Small children notice that too. They see: Oh, but you're not completely Dutch. […] I think that especially young children - who don't yet realize that it's not nice to behave like that - they'll do something different with that and then be unkind or mean because that person is not like them. So then, in primary school, I found that difficult. I often thought, if only I were Dutch, if only I had blue eyes and blonde hair. I remember when I was fourteen, I was with a friend of mine, we were texting and I started talking about my appearance and he asked, okay, what would you like to change? Some people would say, oh yes, I have a spot here, I want to get rid of it, or I want to have my nose changed. And I said to him, I just wanted to look Dutch […] (NL4, Dutch, 21 years, non-binary, recurrent)  At most – I don't know if it was necessarily lonely, but I was just a little different from the other children in my village because I was born in <city in the Netherlands> and my parents were from <region in the Netherlands> and quite clearly a little different too. Uh, also half Norwegian, so I stood out a bit in that respect. But I also had a few very good friends, so uh. It wasn't pure loneliness, but I did notice that I was a bit different. […] In Norway, I was a bit different, but in a way I also belonged in the same way. (NL5, Dutch, 22 years, male, situational)  And then I came back there [to the US] […] and everyone would seem American or international, but both these groups were really, like, specific for me in how they behaved, in how they talked. […] I feel like, just… cause I’ve spent most of my, like, my life in Poland and the culture is different. So, it’s, like, fundamentally something that makes me different from – from people in the US… […] and I kind of, like, sometimes I would have to, like, stop what I wanted to say, cause I was like, ‘Oh, they wouldn’t get it.’, or ‘Oh, it wouldn’t be funny for them.’, you know? (PS5, Polish, 29 years, female, situational) |
| Sexual orientation | I mean, based on my gay identity, I often feel different and disconnected from people. I mean, I had my first realization at the age of 13, and I accepted saying that I'm probably not straight when I was 16. I think there have been many, many times when I felt like a misfit in the 12 years between the ages of 16 and 28 - I consider those 12 years as the period when I felt like a misfit, when I couldn't fit in, and felt left out. Overall, this feeling seems to have permeated my life. (T1, Turkish, 28 years, male, recurrent)  Actually, I still feel this sometimes at the office. I remember this one incident! Everyone was chatting during lunch about weddings. Who's getting an arranged marriage, who’s going for love marriage, honeymoon plans, they were even discussing baby names. I stayed quiet. I was smiling, but deep inside I felt completely cut off. I thought, if I said I had been in a relationship with a woman, the entire atmosphere would freeze. […] Yes, there’s a vivid memory from school. After 11th-grade summer break, all the girls were talking about their crushes and boyfriends. I liked a girl, but I couldn’t say anything. When someone asked me who I liked, I lied… just named some actor. Deep inside, it felt like I was cheating myself. At that age, confusion is normal, but when your truth feels unacceptable, you start drifting away from yourself. (D8, Indian, 25 years, female, recurrent)  Um, I’m also gay. So, that was also a part of growing up where I did feel very alone. Um, and I remember the months before telling my parents and telling my friends and being so terrified of, like, what would happen and thinking ‘It’s just me. No one else must feel like this. It can’t be this hard.’ […] Yeah, I think being gay, that’s such a - like, a big thing. […] You see it – like, used to see it in tv-shows or films that obviously other people are gay, but you still feel like it’s such a big thing to yourself. Um, and you know that you’re different. And, like, you see your friends […] going to prom at school. Like, seeing the boys ask girls to prom and me just being like, ‘I –‘ - because obviously it was very special for them, um, as, like, heterosexual people. It was kind of, like, cute and romantic. But for me, it was very much ‘I can’t really have that’ because there’s not - I can’t really approach one of the lads at– well, especially not the school that I went to. There was no way that I could approach one of the guys and be like ‘Do you wanna go to prom?’, you know? […] Because I still do feel different, like, when I go out. I’ll – I’ll not want to go to a club which is probably gonna make me feel uncomfortable. Or a bar that’s not really the setting that probably would be safe for, like, a gay guy to go to. (PS8, British, 22 years, male, recurrent) |
| Gender norms | Well, I was also one of the few people who was assigned female at birth who found video games interesting. I thought that was very strange, because I wondered why the other girls didn't want to play with me. All my friends were boys, and because of that, they probably thought I was strange too. They thought, “Oh, she's in love with all four of those boys”. No, no, that's not how it works. (NL4, Dutch, 21 years, non-binary, recurrent)  I mean I wanted to play basketball, so I joined the team. And, um, in my school, like, girls that played basketball was not really a thing, uh, and I-I-I had to leave, like, the year after, because I didn't feel comfortable. […] I mean, in school, girls wouldn't- wouldn't join the basketball team. So, there were- I remember there were only two girls plus me, uh, and it was kind of a mixed group and- and I would see with other schools that would play in the- in the school league that, um, there were no girls. […] Um, but it was more, like, in my school, like, when I go back- go back to the classroom, you know, they will ask, you know, “What do you play?” and my answer will be basketball instead of volleyball or- or gymnastics. (PS2, Venezuelan, 25 years, female, situational)  Because, you know, when we look at it from the perspective of Turkish norms, in a [city in Turkey] school where there are rowdy people and where you can witness more toxic masculinity, being someone who attends a middle-class college, you can see a lot of toxic masculinity there. Imagine a boy in that class. He would come to class every week with cupcakes and constantly talk about Broadway musicals - you know, weird. Why? This kid is weird. (T1, Turkish, 28 years, male, recurrent)  Just people going to the gym all the time and playing soccer and sports and doing masculine things. Um, so, I think the feeling of not fitting in was mainly during um gymnastics. […] So, it was a lot of comments like, oh, yeah, “the gay guy” or whatever. “Oh, yeah, he's really feminine”, stuff like that. (NL1, Dutch, 26 years, male, situational) |
| Neurodivergence | Simply because I have different life experiences that I know my friends cannot understand. For example, I am transgender. I am autistic. […] I've always joked that if you're autistic or have ADHD or are neurodivergent or different in some way, you don't need to see a psychologist, you need to be in Year 4 and talk to popular people. Because no one notices that you're different as quickly as a primary school bully. […] Also because of autism, for example. I know that I think differently than the average person, but knowing that I am different from others and feeling that I don't belong with the average person has little influence on my self-image now. (NL3, Dutch, 22 years, transgender male, recurrent)  It took a long time for me to understand how I should behave in society. It wasn't because of my family or anything like that. Currently, I'm sort of attributing it to something else, I had intense hyperactivity. I used medication for about seven years. At that point, children's minds are like - they act impulsively and do whatever they want. Then they realize that they shouldn't do it. It's like putting your hand on fire. You don't know it's wrong or harmful until your hand gets burned. I understood those things relatively late. […] I didn’t fully understand that or understand social cues. Maybe even now, I feel like I still don't understand them very well, although it’s happening less than before. Also, during the early years of university, I couldn't understand them, I guess. (T10, Turkish, 26 years, male, recurrent) |
| Preferences and perspectives | Hobbies are just one aspect. Actually, what makes up a person? A person is made up of their hobbies, their personality, their thoughts, their values, their understanding of the world, all these things. That's what makes a person. So it's not just hobbies. For example, if I want to talk to them [others] about thoughts and about understanding the world, I find the gap is very big. (HK2, Chinese, 19 years, male, recurrent)  And the theme [of the party] was neon and, um, everyone was, like – felt, like, very different from me. Like, […] what they would like to do at a party and, like, playing beer pong and, um, just, like, behaving in ways where, normally, um, like, I don't like to do or, um, like, I don't, yeah. (PS4, German, 26 years, female, recurrent)  So, here’s the situation—my mother joined a charity organization. However, its core philosophy is actually based on Buddhist principles. They advocate for beauty, joy, love, and awareness. They hoped to integrate these principles into early childhood education, believing that if every child embraced such a positive outlook—if we cultivated an environment where everyone expressed beauty, joy, love, and awareness as a way of life—it would greatly benefit early education. Or so they thought. I have participated in these events two or three times, not only as a student, but also as a volunteer. I served fellow students and trainees, striving to be more cheerful and optimistic than the students themselves—or rather, more attuned to the environment. I felt like I was doing a good job, but at the same time, I didn’t feel fully integrated into the program on a deeper level. My underlying belief is that this kind of growth approach isn’t necessarily suitable for every family. If a child's family environment is stable—meaning their financial situation is fine, and their parents have a good relationship—then yes, growing up in this kind of positive atmosphere can shape them into optimistic individuals who look forward to the good things in life. But for some families, especially those with serious issues, I saw many children who came from difficult backgrounds. Some of them joined this program hoping it would help save their family or children. However, I actually think that, in cases like these, this approach might end up ruining them. (HK6, Chinese, 24 years, male, recurrent but not throughout life)  (for additional quotes, see *Interests* and *Opinions or values* on p. 15 below) |
| Achievements, skills, or level of intelligence | My self-discipline wasn't so great before, so there could be a sense of disparity. Um, like when I was in my first or second year, I went on an exchange for a semester abroad, and the students who went with me were all seniors. My self-discipline wasn't great, and I didn't do too well academically when I came back, and that increased the feeling of disparity, kind of a psychological distance from my classmates in the same department. (HK3, Chinese, 24 years, female, recurrent)  I have also felt left out academically as well, especially it has been prevalent here. All the people in my class… Since you don’t get into a university without being good in studies, so I have always felt that I don’t deserve to be here and that my classmates are doing so well while I can’t keep up with everything, so that has also made me feel left out. (D1, Indian, 21 years, female, recurrent)  So, it’s my master’s journey! Since day 1, umm… the kind of people I met, everybody in the class – everybody was so smart, theek hai? ( = okay?) I felt so stupid. I felt so dumb. So that was one aspect that I felt that I don’t fit in. These people have earned their way to get here, and I am probably not really smart to be here. (D2, Indian, 22 years, female, recurrent)  When I see them [secondary school mates], especially when they come home for vacation. It used to make me feel that, if I have also gained admission, I would be able to roll with them better. They dress better. (NG4, Nigerian, 24 years, male, situational)  I don't want to sound elitist, but the average educational and intellectual level at my primary and secondary school was a bit lower than mine, which meant that people thought it was strange that I could already read in Year 3 [age 6]. And we had... in Year 6 [age 9 or 10], people were taught the alphabet. So it was already the case that people simply didn't understand what I was talking about because I had too large a vocabulary. […] My parents both finished secondary school at “mavo” level [i.e., a lower level] and that's it. As a result, now and then, when I talk normally, I hear things like, ‘Oh my god, since you've been at university, you suddenly talk in such complicated ways and I just don't understand you anymore. Can you talk normally?’ While I feel like I talk quite normally. So then I have to actively simplify my language for them and speak more slowly. And I have to actively make sure I don't use theoretical concepts, which makes me feel like I'm trying too hard to be someone they understand and like, rather than just being myself with my family, so to speak. (NL3, Dutch, 22 years, transgender male, recurrent)  When I was quite young, my parents had a psychologist or something like that who said, “This child is gifted”. Um, you could see that in my behaviour or how I did things and thought about things. I was always one of the best in the class. I was one of the quickest to answer questions, I was one of the quickest in tests. I always got high marks. So I had interests that were a bit ahead of my age. […] But that's also one of those things that, when I look back, I think, 'Gosh, I just felt left out because I was cognitively a bit further ahead than my classmates or my peers. And that's why I connected so much with people who were fifteen or sixteen, while I was twelve. (NL4, Dutch, 21 years, non-binary, recurrent) |
| Personal tendencies or personality characteristics | However, agar isko bhi side rakhe (=if we keep these things aside), there is my family that has never understood me. Again, you know how it is in Indian families, they don’t particularly talk about things, right! And I am not a person who cries, but in the past whenever I have cried due to something that was really concerning or something that was heavy for my system and I wanted to relieve it, all of this has somehow never been received well. I have been told things that how I am exaggerating or ‘drame kar rahi hai’ (=she is doing drama / being a drama queen). (D2, Indian, 22 years, female, recurrent)  And I would say, like, other, like, ways of feeling different is just like: I am more - I’ve learnt to be rational because […] I used to be super […] subjective. Yeah. I think that's the word. Like, you know, like, my friends are more rational. They're just doing, like, what they think is logic and, like, what they think is fine. And I'm not really always doing that because a lot of times I think more with my heart or, like, with things. And they're just, like, doing what's logic and there we clash as well. (PS3, Venezuelan, 23 years, female, situational)  (for additional quotes, see *Introversion* on p. 16) |
| Appearance | Physically, I am a big guy, and from an early age, I stood out from the rest of the class, tall and burly, in the back row. We were two such kids who attracted a lot of attention, even among our peers. So, it started with a physical separation. (T1, Turkish, 28 years, male, recurrent)  Because of my unique body, I was kind of fat, and I was laughed at first. (HK11, Chinese, 24 years, male, situational)  I have piercings and green hair and a slightly different clothing style than average, and I've had these interests for a very long time. It's only now that I dare to express them. But when I did reach for those things as a child or young teenager, just for the things that made me happy, I knew: oh, this isn't really part of it. This isn't really how it's supposed to be, and that makes you different. And there's little that can be as bad in a conservative high school as being different from the rest. (NL3, Dutch, 22 years, transgender male, recurrent)  Cause I was living alone. I had to make my own food and all. You cannot expect me to dress up for a 9 am class in the morning. […] Occasionally, I wear something nice. Not all the time. They do it daily. (D3, Indian, 20 years, female, recurrent) |
| Developmental timing | So, I was a bit more childish in a way than most people for a long time. […] I didn’t really wanna grow up as quickly as society wants or as quickly as maybe others wanted. Because I kind of realised the liberties that I still have being a child or being seen as a younger- uh, a young one. (PS7, German, 28 years, female, recurrent)  However, recently, for example, I have some friends whom I'm not very close to but occasionally chat with. When I'm with them in a group setting, there are times when I can't find the right words to contribute to the conversation. For instance, discussions about marriage, having children, or being in a serious committed relationship with someone, topics where I don't feel I am at that stage [of life] yet, and the conversations continue in that direction while I struggle to find my place. (T5, Turkish, 25 years, female, recurrent)  But that's also one of those things that, when I look back, I think, 'Gosh, um, I just felt left out because I was cognitively a bit further ahead than my classmates or my peers. And that's why I connected so much with people who were fifteen or sixteen, even though I was only twelve. (NL4, Dutch, 21 years, non-binary, recurrent)  Hmm, I do feel more like my peers. I can relate better to their needs and motivations. But I still have the experience – I had it much more as a child – that I actually noticed that I have better conversations with adults than with my peers and feel more at ease. I feel – um, I can relate to them more, let's say. (NL6, Dutch, 22 years, female, recurrent)  And after my father passed away when I was younger, I think that also made me grow up faster than others. (D9, Indian, 27 years, male, recurrent) |
| Relationships | I already didn’t have a mother figure to look up-to, so in general I always felt that ‘Oh! There are things that, you know, moms and daughters share and whatnot’, but in that respect also I felt missing; like people in school… I will give you a small example of school. Like, I used to not take tiffin, so people used to take tiffin in school and say that ‘oh! Tum tiffin kyu nahi leke aayi ho? Kya tumhari mummy ne kuch banaya nahi kya?’ (=Oh! Why don’t you bring lunch box with you? Does your mum not cook for you?), and that point of time, I was so small and I did not know what to tell and the second thing was I did not want to tell as it was my family matter. Umm… but when they used to share about their families like how it was ‘normal’, so uhh... I used to think ‘why not me? Why did I not get that? Why did I get such a hostile household?’. So, again, there was a void in that respect. (D2, Indian, 22 years, female, recurrent)  Like, it’s also weird cause you don’t fit into, like, the idea of, like, having a family, cause I don’t anymore. Like, I just have my brother. And um, like, I was in the hospital recently to get my tonsils removed and then my nurse, she was like ‘Oh yeah, is your family going to come and pick you up?’ Or, like, it to- ‘Is your family almost here?’, something. She was already thinking that my family would come and pick me up. So, it’s already kind of like [participant makes noise to signal discomfort], you know? It’s, like, a bit awkward. […] And the same goes with, like, if there’s, like, mother’s day, or if there’s, like, at work we have like a family day and I’m like, which family? Like, um... I’m happy to have my friends, though, cause they are like a very good support system in this. Just, I’m very happy with that. But um, but yeah, it’s weird to not have this support system that has known you your whole life. Uh, that can help you with, like, growing up, and taxes and things like that, yeah. (NL2, Dutch, 26 years, male, recurrent)  When I am with my ‘couple’ friends, I am like a third wheel in between them. I can see that they are having a great relationship and I am sitting alone in the corner. (D4, Indian, 30 years, male, recurrent)  Another time I felt lonely was during Rakhi [Hindu celebration in August] —when others talk about their sisters tying rakhi [tradition where sisters tie an amulet around their brothers’ arms to protect them]. It hurts me because I don’t have a sister. […] Like, all my friends have sisters, and I don’t. It’s the same pattern—when you lack something, and it’s not in your control, you feel different. (D5, Indian, 27 years, male, recurrent) |
| ***Situational causes*** | |
| Older age / maturity | Well, quite often when I was young, but not anymore now. (NG6, Nigerian, 19 years, female, situational)  I still experience it to some extent. But it doesn't happen as frequently anymore. I guess I've learned to manage it a bit, I don't know. (T6, Turkish, 26 years, female, recurrent)  I felt very isolated with that. Sometimes [I] felt so unique and like - it was: nobody would ever experience anything like it. But I think, I mean, growing up, obviously, you saw and learned that other people do, in just very different ways. […] Um… but I definitely think, now I’m a lot older, and I’m a lot more like open about things. (PS8, British, 22 years, male, recurrent) |
| Little familiarity with group | It’s less frequent now, but whenever I’m with new people or in formal settings, that feeling resurfaces. (D8, Indian, 25 years, female, recurrent)  It happened in these kinds of settings. When I met people outside my own circle. […] It happens a bit when I enter environments with people I don't know. (T7, Turkish, 20 years, female, situational) |
| Moving to a different culture | Um, and um, right now, because of a different country, different language, it’s – it’s way harder to fit in, so-to-speak. [I: Yeah.] So – so, when you know that you already have your friends and the way - especially the way you express in your own language and then you come to another country, it’s way harder to - to be yourself, you know? Um, so, I think would be the those - the two situations where I felt that I didn’t fit in. (PS10, Colombian, 27 years, male, situational) |
| ***Contextual causes*** | |
| Contexts with low tolerance or understanding for deviations; social homogeneity; narrow or strict norms | And there's little that can be as bad at a conservative high school as being different from the rest. (NL3, Dutch, 22 years, transgender male, recurrent)  I used to think – like, used to think, would it be - would my outlook be different if I lived in a different area? Um, because I’d live in a very, like, working-class area, and it was very white, and I would definitely say, like, a bit of rough place to grow up. Um, and I would just think like, would it be different if I went to an area - a little bit more, like, affluent. (PS8, British, 22 years, male, recurrent)  It’s less frequent now, but whenever I’m with new people or in formal settings, that feeling resurfaces. (D8, Indian, 25 years, female, recurrent) |
| Contexts with few opportunities to leave; small communities | So, the school that I went to was my second choice but had probably about 300-400 students. And my first choice - the one that I wanted to go to, was a lot bigger. And they had about 1000 students. And I always thought: Would my thoughts and feelings be different in a bigger school, where there’s probably more students like me? Where that likelihood of being around all this is higher. I definitely thought about it at school, but then, when I went to college – um, I went to a college with, like, 1500 students and that was completely different. That was eye opening. […] But definitely, while I was doing my 11-16 years old education, that’s what I used to think. Like, ‘Would it have been better in a bigger school?’ […] And then, I think that magnified and got bigger when I’d started university. Um, because again, there’s even more people. (PS8, British, 22 years, male, recurrent)  Due to financial reasons and anxiety about my future, I can't quit [the job] right away. I want to move towards a different area, but I feel isolated because everyone around me seems satisfied with their work in this field. […] As I said, we live in a country where the future is uncertain. We need to take slow steps. I don't think I will be supported if I act impulsively. Also, I think differently about this than my family. When my thoughts don't align with theirs, they would brush me off or discourage me. (T5, Turkish, 25 years, female, recurrent) |
| Environments fostering comparison | So, because of that, you always compare yourself - I mean if you keep in this competition behaviour, you always compare yourself to the others, um, even though it’s something different. For example, if I like, I don’t know, paediatrics, and the other one likes psychiatry - even though if I say it that way, I would see that person that likes psychiatrics still as a competition. […] So - so, for example, when you see in - I don’t know, in Insta stories or in Facebook - that everyone is posting that they are showing everyone that they’re in – already in medical residency, […] I think it’s more- not to validate themselves, it’s more to show the other ones that they’re already in that part of the medical pathway, so to say. [I: Yeah, yeah.] So, that, like, stressed me a li- It stressed me and also makes you like, ‘Okay, why are you not- why are you not still there?’ (PS10, Colombian, 27 years, male, situational) |
| ***Personal causes: increased sensitivity to not fitting in*** | |
| Insecurities and high self-awareness | I feel like that feeling of fitting in more came also from factors that weren’t external. Like, from - coming from other people. But just like, I - I don’t know, somehow internal. [I: Yeah.] Like, once I felt more, like, settled in that place, I felt - or at - at least it felt easier to try to fit in. At least be doing tho - those efforts to fit in. (PS5, Polish, 29 years old, female, situational)  I’m afraid of judgement of others towards me. So, that this anxiety- so, if I do this or if I sit on the floor now, what would people think? Do they think I’m weird? Because I already felt weird. (PS7, German, 28 years, female, recurrent)  I still feel like, even like, there's this one guy in my programme who's really nice and another girl who's really nice and I think that I still have lots of negative thoughts and feelings of, that's not so much loneliness, it's more just like self-criticism, and I think ‘Oh, I'm not as good as them’ or ‘I'm not as smart, I'm not doing as well, they, they seem to be so much more in control of everything’. (PS12, US American/Belgian, 29 years, female, recurrent)  And like, I was also new in school and I wanted to fit in as well, um. […] And I didn’t feel that pretty, I was a like I- what I remember, um, I was- I was a bit fat. Um. That’s- like my body changed a lot in 4th and 5th grade, from what I remember. But third grade, like, I just didn’t feel pretty. Like, I didn’t feel that I belong in such a big school. I was new and I don’t know. (PS2, Venezuelan, 25 years, female, situational) |
| Desire to fit in | And like, I was also new in school and I wanted to fit in as well, um. (PS2, Venezuelan, 25 years, female, situational)  I think people [easily feel different] who care too much about what other people think. (HK12, Chinese, 26 years, female, no experiences of feeling different)  At the campsite, there were other friends of mine who weren't part of that organizing committee, and they felt it didn't matter. […] I wondered if the others felt it didn't matter [that they were not part of an organizing committee], but only I cared about it. Am I unable to just live on my own? My friend said if I didn't care so much about it [being part of the group], I could have smoothly integrated with the group, like just walking a few steps faster to catch up and chat with them. […] When I see some people don't care about these things, I wish I could naturally feel the same way. For example, if no one is playing with me at the moment, I should enjoy my own time. But I don't enjoy my own time so much. I can enjoy doing nothing, lying in bed, but I need someone, like my mother, to be by my side. (HK1, Hong Konger, 19 years, female, recurrent)  You feel left out already, because you want to be like that person, but you are not up to the standard of such person. (NG9, Nigerian, 21 years, female, situational) |
| Stress and illbeing | If I'm in a bad state, the frequency of feeling "left out" will be higher. When I'm in a good state, I can resolve those emotions on my own. (HK1, Hong Konger, 19 years, female, recurrent)  Also because it was a bit of a—well, side effect of me not being in a good place emotionally. (NL3, Dutch, 22 years, transgender male, recurrent)  At that time, I wanted things because my personal situation wasn’t the best. So, I wanted to feel welcomed at least cause every other place I felt that I was being thrown out of the situation. If it makes any sense. (D2, Indian, 22 years, female, recurrent)  Well, in my family, I have to say that at a certain point I did become a bit of an odd one out, but I think that was mainly because I had a lot of problems. And I just felt really bad about myself, which meant my parents didn't really know what to do with me. (NL6, Dutch, 22 years, female, recurrent) |

**Table S2.2**

*Additional Quotes by Participants with Recurrent Experiences of not Fitting in*

|  | **Participant quotes** |
| --- | --- |
| Indicators of recurrent feelings of not fitting in | Actually, [I’ve been feeling different from others] for as long as I can remember. That is—yes, feeling that connection is something that has only happened in recent years. I feel like I don't belong or that I'm left out almost every day. (NL3, Dutch, 22 years, transgender male, recurrent)  Yes, on an everyday basis, something or the other happens that makes me feel like the odd one out. In daily life situations, in everyday moments, feeling like the odd one out is a constant feeling for me. […] And I feel that when I’m around people, I always end up feeling like the odd one out. (D5, Indian, 27 years, male, recurrent)  Yeah, I think this is something that, throughout my life, I have felt quite often. Um, like, sometimes it's more of a general feeling. Sometimes, it's more a really specific- like, in a group and then you're like: “Oh, I don't fit in here.” But sometimes it's also more like a vague, like, “Oh, like, I don't fit into, like, society.” or something like that. […] In a group context, [I have experienced this] quite often. Pretty much as soon as there is a group setting - even if it's my own friends - I would feel like this. (PS4, German, 26 years, female)  That's a good question because I don't know, uhm, I think I've always, like, forever kind of felt different and lonely. […] I think I've always felt weird and different […] ...as you said, it's still not, like, resolved, but uhm, I think that I've connected with a few people that I should probably reach out to more often. (PS12, US American/Belgian, 29 years, female, recurrent)  My entire life. […] Um, I’ve always felt different my entire life. (PS6, Venezuelan, 24 years, female, recurrent)  Ah! [I have felt different] A lot. A whole lot. […] In fact, [I feel different from] everybody. Sometimes, family, colleagues, and people I know. It could just be a random person on the road. (NG10, Nigerian, 19 years, female, situational)  Overall, this feeling seems to have permeated my life. (T1, Turkish, 28 years, male, recurrent)  Honestly, including my university years, almost always. I can't say I never (felt connected), that would be an exaggeration maybe. (T10, Turkish, 26 years, male, recurrent)  I feel it very often, every day. (T2, Turkish, 26 years, female, recurrent) |
| **Social norm related to …** | |
| Interests | Yes, even during school days I felt that way. While everyone ran to play cricket, I used to go to the library. I preferred drawing or poetry. (D7, Indian, 28 years, male, recurrent)  Most people like specific movies or series and, um, know a lot in common, [I: Yeah, yeah.] or have a lot in common regarding these- type of themes – topics, what I really don’t have. So, I have no idea about uh many- most of the series, certain books that everybody read but not me, um, tv-shows or movies, that are mainly related to action and horror, which is something I just - I am not into at all. And that already… like, when through the - also music, for example. During high school, I didn’t listen to the music that other people listened to. […] I felt different in many ways. So, number one, interest. So, people started to go out, dancing, having boyfriends, drinking. And I- I would have loved to go out dancing, but not disco, but I would have loved to do partner dances. Like, I love now to dance bachata, kizomba - these are the things I really like. But back then, in that village, Southern Germany, it didn’t exist. So, it was basically beer parties. I didn’t like beer. So, I didn’t like - I didn’t see a sense in getting drunk, for example. […] I was always interested in and grateful to be able to go to school. I don’t know where this gratefulness came from, but I was always enthusiastic, I always wanted to learn, which is a not common thing at all among teenagers. […] I had a different attitude towards school than other people. So, when people complained: ‘Uh, I really don’t wanna do - I don’t want to learn now, dadada…’ And I couldn’t really support them in saying this. […] And then, I was interested also in more spiritual things or - Yeah, kind of, let’s say, spiritual things, or also indigenous cultures. Like, stuff that is not very a mainstream teenage interest type of thing. (PS7, German, 28 years, female, recurrent)  (for additional quotes about deviating from *Gender norms,* see p. 6) |
| Opinions or values | So, they all have this kind of mindset of living the same kind of life entirely, like taking responsibilities at home and just only looking for themselves only. Okay? So, my idea is a little different. I want to be free, kind of liberated. Kind of away from responsibilities. I do whatever comes in my mind. But they are like no, no! They are very much concerned about every step they take, their concern about money and family. How to take things in their own way. So, they have a very different ideology than mine. (D4, Indian, 30 years, male, recurrent)  So, even if I, like, just went to the city and saw someone, like, littering or buying fast fashion or whatever, I can get this feeling [of not fitting in with others]. Things that don't align with my values, for example. (PS4, German, 26 years, female)  My parents became parents quite young and weren't very financially stable at the time, which meant they used to be quite left-wing and open-minded, and those are the ideals they raised me with. Almost socialist, in fact. But now my parents earn quite a lot and are big and strong and mature and tough, and they've completely reversed their political compass. But that didn't happen to me. So now there's little overlap there. (NL3, Dutch, 22 years, transgender male, recurrent)  (see also *Religion or religiosity* on p. 21) |
| Life experiences | Well, to be honest, I feel this way in most new environments. Currently, due to some difficulties I've been experiencing in my life, I generally often think that I'm different from everyone else and alone, as if there was no one around who can understand me. (T6, Turkish, 26 years, female, recurrent)  Also, mentally, I’m often thinking about home—about Ma, her meds [medication], or next hospital visit. My mind is not 100% free like theirs [other people’s]. So, I act more… withdrawn. (D9, Indian, 27 years, male, recurrent)  Um, I had a bit of a – a bit of a weird childhood. I didn’t really get on with my mom’s partner. Um, and it felt like I was the sort of only person going through that, um, even though, like, friends at school would say like: ‘Oh, I don’t really get on with so and so’, and ‘I don’t get on with them’ or whatever. I felt very isolated with that. Sometimes felt so unique and like - it was: nobody would ever experience anything like it. […] I think, my second year of university, um, my mom was very ill. And that was - it was awful. And it was to do with, like, her mental health. And I, j- because I am the eldest, I had to sort of, like, deal with it all. […] And, um, I had to deal with it, like, all myself and then, sort of, give out a much toned-down version of what happened to everyone else. Um, and I didn’t deal with it very well. And um, then I got assaulted in the summer this- of the same year. Um, I just got robbed at a post office at, like, 11 o’clock at night. Um, and then didn’t deal with that very well. […] I felt like I had so much traumatic and emotional baggage, and it was just me again. And I felt so li- I felt everyone else was just living this perfect life and nobody else had got this massive stress and I did really feel different. (PS8, British, 22 years, male, recurrent) |
| Introversion (less sociable, less talkative, quieter) | One is that I don’t interact with people a lot… even in school where people used to go out and socialize, but I never did that. […] Now that I have grown up, I have realized that it is just a part of me, that I don’t socialize much. Also, throughout my life, I have been an introvert, but the people in my family have questioned me on ‘why are you like this?’. And even the society does not lets introvert be in peace and in comfort zone... So, that made me feel left out too. (D1, Indian, 21 years, female, recurrent)  I’ve always been a bit quiet, observant. People thought I was “too serious”. […] in a group internship workshop last year. Most people were very outgoing. They were making jokes, doing group tasks with high energy. I participated, but quietly. Some people even asked, “Why are you so silent?” I didn’t feel like I fully belonged. […] I don’t open up quickly. […] I always feel like I don’t know how to act “normal”. I’m polite, but I don’t fit into casual social groups very easily. Like once my mami [mother’s sister] was forcing me to talk to my cousin and her friends in a family function. I did not wanted too. But, since she was an elder to me, I went to that group for barely 2 to 3 minutes and came back to my seat. I can’t talk back to elders. It's hard. […] (D9, Indian, 27 years, male, recurrent)  I think I’m a bit introspective, and I don’t feel that sense of belonging in every group. […] A few months back at an office offsite party. Everyone was enjoying drinks and loud music, and I was in a corner… just observing. It felt like I was present, but didn’t belong. […] My office team — they were all very outgoing and extroverted. Their bonding was at another level, and I couldn’t align with that flow. (D7, Indian, 28 years, male, recurrent)  I've always felt a bit like an outsider in the sense that I just don't fit into a group very well and I don't get along very well in larger groups either. Sure, I can get by, but I become very quiet and I don't really like it. I'm more comfortable in one-on-one conversations and generally prefer to be in pairs or small groups, but that was also just a feeling. (PS14, Austrian, 27 years, female, recurrent) |
| Way of thinking, perceiving, or feeling | And I think that’s something else that used to make me feel different. Like, the way that I think, um, and that - Sometimes the way it sounds in my head sounds perfect, and then when I say it out loud, I think: That makes absolutely no sense. Um, or, like, being overly interested in something. I used to think, like, ‘Is it just me? No one else is liking this book?’ Um…[…] I still do feel different because I know that I am different and that my brain works in a different way, but um, it’s kind of um- it’s kind of nice cause it wasn’t - it was kinda like: This is who you are. (PS8, British, 22 years, male, recurrent)  So, we are having this class discussion, and the lecturer raised the question. I cannot remember the question. Everybody answered to the best of their knowledge, and I also want to answer, so I stood up to also make my contribution. And the lecturer said, "what are you saying?". To me, what I was saying was what I read. Aside reading, it is something I felt was correct. So, my mates were like, “This is what we are discussing, this is what you are saying.” […] The way I think is different from the way others do. (NG5, Nigerian, 24 years, male, recurrent)  I was more of an introverted person who also felt things very strongly, could feel music very strongly, just generally felt things very strongly and was also very perceptive of other people. […] I just felt very, very profound […] even as a child, I often felt somehow a bit more profound, and I would say that this is still partly the case. […] I just experience life very strongly, also through my psychoses, of course, just very strongly, very intensely. So, I've always experienced things very intensely and that's why, yes, I would say that maybe some channels were somehow more open than in other people and I was perhaps more sensitive in that way. (PS14, Austrian, 27 years, female, recurrent)  Um. It was mainly the way of learning [that was expected at school] that made me feel very confused about what was going wrong, because... Um. [silence] Well, it's actually quite a simple situation, but I just had the feeling that it was very clear to a lot of people what the intention was. And I felt that I had a different approach to learning. Um, and that, yes, that was very confusing. [both laugh] And I didn't understand why it just wasn't working, because I was trying really hard. And yet—there was some kind of invisible, um, something I was missing all the time. (NL6, Dutch, 22 years, female, recurrent) |
| Diagnoses to describe vague recurrent feelings of being different | It still keeps bothering me. It's just strange that all teachers and even the headmaster of the school felt that it was something about me. […] And I know that, at that time, my mother - she didn't really dare to admit it to me - but she also felt it was because of something about me. She did have me sent to all kinds of training courses or to the child psychologist. […] I later did try to get uh neurological testing, but that - well, that yes – also nothing much came out of that. (NL6, Dutch, 22 years, female, recurrent)  (see also quotes for *Neurodivergence* on p. 6) |
| Treatment by others | I don't know where to start, but I think I've always felt weird and different and like in primary school I would get made fun of sometimes and I never really felt like I had a best friend or like I fit in. And then in - in secondary, so in high school, there it was a bit different, because I had a really close group of friends. But I was also like in youth groups and, like, kind of like the scouts, but slightly different. Uhm, and I always felt like, I was weird and I, I would try to, like, act like others, but it didn't quite work. [...] (PS12, US American/Belgian, 29 years, female, recurrent)  You know as a student, I am currently doing my teaching practice as I have earlier informed you. So, that particular day, we were like three to four people that did not come to school. The following day, when I got to school, and the four of us normally go together. So other fellow students who were also undergoing the training asked the others why they were absent in school the previous day. But, none of them asked me why I was absent as well. I felt that my absence did not worth it. Like, we all knew ourselves [each other] from the first day when the exercise has started, and we have all been interacting together, then why the difference in the treatment? I still wonder on the bias. […] Like when I am not being treated as they treat others. When things happened, and I am not being noticed. Interacting with others while not interacting with me. So, sometimes, I feel like, is it that I frown my face, or I don't have a good aura about myself? I don't know. I just wonder. […] My colleagues like those that we are doing teaching practice together, not only that, but also the teachers. I have a course mate that we are having our TP in the same school, as I said, this is my second TP, it was this same school that we both used for our first TP. My friend is not being treated as a stranger; however, it is the opposite for me. They call him more, use him more. If the teachers need to pass a message to other TP students, they call him. You know, I was like "we did this thing together. We were both here the previous one as well as now", why are they not engaging me as they engage him. […] There was a particular day that I was travelling. When I got to the car park, I met a lady there; or was I the first to arrive? I could not remember. That day, there was a rush for buses. So, we were there together, not that we are really conversing, it was more of me asking her questions like ‘where are you going’? Maybe because I always have this feeling that I am different from others. So I tried to engage her, even, I know that I overdid it, I tried to make an effort to win her into conversation. This lady's response was just monosyllabic-No/Yes answer. Then, another lady came to join us, she was also going to the same location as we were. We took a cab, the three of us occupied the back seat. Then, normal conversation in a travelling cab, people move from politics to economy and all. I was also contributing to the conversation, then I realized that the lady that was previously giving a yes or no answer, was conversing so well with the third lady that later joined us. I was even trying to be part of their discussion, but, she just gave me a sort of attitude that made me feel "let me just shut my mouth". I was just like, we met earlier than this other persons that she was communicating freely with. I was even the one encouraging her that more buses and cars would still come while at the park. So, I just feel like if someone and I get to a place, the other person will be more preferred than me. (NG3, Nigerian, 26 years, female, recurrent) |
| Feeling or being signaled to be different in family | ***Participants with recurrent experiences of not fitting in:***  My parents... of course, they don't have similar personalities to mine. Otherwise, I wouldn't feel lonely, right. They are very different from me in personality. […] They have their way of expressing love. […] But I can't understand it. (HK2, Chinese, 19 years, male, recurrent)  Let me say my family. In my family, we are three boys and two girls, but the way our parents interact with us, I feel they treat me like I am different. […] Daddy is an evangelist. And me, I am not a religious type. But others in my family are this over religious persons; everything is church, church, church. We had this family discussion few months ago, and it was like “say whatever you have in the mind that could help move ahead as a family, I raised about the issue of religion, and I was shout-up that I should never in my life bring about that kind of matter. And I was just trying to bring this perception that, “it is not everything we do that should be about church, church, church”; there should be some things that we apply common sense to. But I was made to know that in this family, it is either God, church, or nothing. (NG5, Nigerian, 24 years, male, recurrent)  This feeling also occurs within my family. I experience this feeling within my family too. I feel different from them in terms of thoughts and beliefs. Of course, this might be due to a generational gap, but I feel very different in every aspect, and I often experience this feeling intensely, especially in my family and with close relatives. (T2, Turkish, 26 years, female, recurrent)  ***Participants with situational experiences of not fitting in:***  And then, even in- in my family, you know, not- not my parents but in general, there was that idea of, “Oh, girls that play basketball are lesbian.” So, I don’t know, um, so, that was also there and that was- it's- I mean, now that I'm older, I see that it's fucked up, but that’s kind of how it was spoken about. (PS2, Venezuelan, 25 years, female, situational)  I: So, what sets you apart from that group is your higher stress level due to your busy work life, and you think they don't understand that very well, is that right?  T3: Yes, I definitely think they don't understand.  I: Alright. So, generally, who are these examples with? Is it more about your family or people who are more distant?  T3: All of them. I experience this with my family in a different way, too. I experience it with my friends as well. What was the question exactly? (T3, Turkish, 26 years, male, situational) |
| Feeling or being signaled to be different in primary or secondary school | ***Participants with recurrent experiences of not fitting in:***  I don't know where to start, but I think I've always felt weird and different and like in primary school I would get made fun of sometimes and I never really felt like I had a best friend or like I fit in. […] Uhm, and in primary school, for example, like, I was quite good at school and I would get made fun of because of that, but then one of my close friends, who was also really good at, like, everything academic and she didn't get made fun of. (PS12, US American/Belgian, 29 years, female, recurrent)  Yes, I did feel a bit bullied. It wasn't that I was being followed or anything, but I did feel that a lot of people were making fun of me or saying things that were clearly meant to hurt me. And I couldn't do anything about it myself, because I was twelve and didn't understand what was going on in my life. So I found that very difficult. […] in primary school, it was my classmates [who I felt different from]. In secondary school, it was my classmates. It was just a lot of classmates who didn't have the same background, didn't have the same world view, didn't have the same experiences. (NL4, Dutch, 21 years, non-binary, recurrent)  So, the thing is that in school, there was a bullying issue in the beginning. When there was bullying, self-confidence was lost at that time. (D5, Indian, 27 years, male, recurrent)  For example, people in high school used to always say that I was ‘weird’ or they knew me as ‘odd [participant's name]’ (T1, Turkish, 28 years, male, recurrent)  ***Participants with situational experiences of not fitting in:***  Or when the teacher assigned group tasks, everyone had different opinions, and it was rare to find someone whose ideas aligned with mine. I’d want to dive deeper into certain topics, but others wouldn’t see the point. That would make me feel lonely, like no one truly understood my perspective. We weren’t quite in sync. (HK7, Chinese, 28 years, female, situational)  Ehm, well, my most concrete examples, think about just my high school, which was quite a heteronormative environment - especially within my class. Just people going to the gym all the time and playing soccer and sports and doing masculine things. Um, so, I think the feeling of not fitting in was mainly during um gymnastics. (NL1, Dutch, 26 years, male, situational) |
| Participants explain recurrent experiences of not fitting in by past experiences of not fitting | Now at Lingnan, I feel a distance from other classmates, which is a very historical issue. […] My parents... of course, they don't have similar personalities to mine. Otherwise, I wouldn't feel lonely, right. They are very different from me in personality. (HK2, Chinese, 19 years, male, recurrent)  And I told him [new boyfriend] afterwards, also: “I felt a little weird because these were kind of repetitive things in my childhood, so youth- like, yeah, teenage years, and now it kind of came back with people I really like. So… [laughs] I was like - I felt a little bad, excluded. […] So, I didn’t fit in in my teenage years and it probably has an effect also now because I never really - I don’t feel I fit in anywhere because, of course, I am - I have been – like, I was born here. […] Yes, other people were mainly people at school, basically, back then. And it also went on to, like, basically - I think this set – this had this - well, this gave me this pre-setting also to feel different. (PS7, German, 28 years, female, recurrent) |
| Less trust in others | So, also in relationships, not being able to trust anybody. Um, that is […] a huge thing. So, that might relate to the not being able to rely on anybody, actually, now thinking about it. Yes, I do not trust anybody, um, and it makes it hard in some situations. (PS7, German, 28 years, female, recurrent)  But this made me think that everyone around me is homophobic in a homophobic country, and even those who claimed not to be homophobic were secretly homophobic. I felt like I couldn't trust anyone. (T1, Turkish, 28 years, male, recurrent) |

**Table S2.3**

*Additional Quotes for Differences in Perceived Norm Deviations and Causes Between Cultural Samples*

| **Social Norm** | **Participant Quotes** |
| --- | --- |
| Getting married at a certain age | They would tell me, "You’re in your twenties, there’s no need to go to graduate school anymore. […] Once you have this job in your twenties, you can get married." […] if they were my peers, we might argue back and forth like friends, which would be fine. But since they were elders, I had to respect them. So, I would just say, "I still want to continue studying and working." Then they would respond, "It's hard to find a job in today's society. Even if you get a graduate degree, it's not necessarily easier to find a job. And now that you're in your twenties, if you don't get married, it'll be hard to find a partner in your thirties. You might as well take advantage of your youth now." I find that really hard to accept. […] At the same time, my parents have never explicitly said that I must get married or have children by a certain age. However, they do make comments like, “Oh, other people are asking—your family has two daughters, how old are they now? Do they have a partner yet? Should we introduce someone to them?” […] But right now, my parents don’t put that kind of pressure on me directly. Instead, they put it on my older sister, who is 30 this year. So, the indirect pressure is on her, but I can still sense it. I feel like if she’s "okay," then by the time I turn 30, I’ll be next. But from my current situation, first, I do have a fear of marriage. Second, I don’t feel that I have everything together yet—my academics and career are not where I want them to be. So, I don’t think marriage is something I should even consider right now. Also, I don’t really understand why people have to enter the next stage of life just because it’s expected. I think someone can live a great life on their own. […] I used to joke about it, and my friends would joke about it too—we’d say this to our parents. And our parents would ask, "What about when you’re old? Who will take care of you?" And we’d say, "Well, the four of us will have jobs and money, and when we retire, we’ll just go to a nursing home. After a lifetime of happiness, even if we end up broke in a nursing home, that’s just what we deserve." (HK8, Chinese, 24 years, female, recurrent but not throughout life) |
| Sexual orientation or not fitting in with gender norms | For quotes, see *Sexual orientation* on p. 5 and *Gender norms* on p. 6. |
| Religion or religiosity (Nigerian sample) | Sometimes I see my colleagues, even as postgraduate students engaging in that [examination malpractice]. Sometimes, some of them tell lie; I hate lying. […] God should just help me as a Christian. So, things like examination malpractice, lying, going for wild parties, ah! No, I am not a part in it. But my neighbors, Jesus! God just need to help me. Sincerely, they are almost done; I need to do something before they go. If it is just by the use of tracts or by playing Christian songs and messages, in order to introduce them to Christ. I need to do something. (NG2, Nigerian, 23 years, female, recurrent but not throughout life)  [I felt different from others] Probably when I was in my service year, I served in Jigawa state, where 99.99% of the population are Muslims. So, it was quite hard for me, but at least, I'm back. (NG7, Nigerian, 25 years, male, recurrent) |
| Generational differences | So, for example, here in Venezuela, it’s uh very common that uh sexuality is very linked to your identity, who you are and that also… […] Um, yeah, it’s cultural shaming, basically, if you would like to show or demonstrate that love, which, in my view, should um… it’s - it’s irrelevant to whom it’s directed or to whom it’s erm given or gifted - it’s just love, right? But here in Caracas, Venezuela, something that is very prevalent is - that it's of course, like, slowly uh slowly changing. I – I believe our generation - and I include you, because I’m aware of your age as well, so below 30 - we uh - we’re becoming more and more open to just: that’s a fact, that’s a fact of life um. However, we- we still – I mean we’re still surrounded, we continually interact with people older than us, whether it is at a job, or in our families, or just day to day interactions. So, I would say that it's most of an issue - more of an issue for people in- in that group. (PS6, Venezuelan, 24 years, female, recurrent) |
| Treatment by others (Nigerian sample) | Last year, Christmas. So my friend said they are going out. I was already on my way to the house when I asked their mom and they said that they have left. So after some time, she called me that I should come and join them, but I couldn't meet up with her. […] My friends were talking about school stuff, but they did not call me. (NG10, Nigerian, 19 years, female, situational)  In my family, we are three boys and two girls, but the way our parents interact with us, I feel they treat me like I am different. (NG5, Nigerian, 24 years, male, recurrent)  (for quotes from different samples, see also *Treatment by others* on p. 18) |
| Different cultural background after moving countries; different language spoken | Because of a different country, different language, it’s – it’s way harder to fit in, so-to-speak. […] Especially the way you express in your own language and then you come to another country, it’s way harder to - to be yourself, you know? Um, so, I think would be the those - the two situations where I felt that I didn’t fit in. (PS10, Colombian, 27 years, male, situational)  I've been studying in English since, I would say high school. Um, and it was when I came here to the UK, uh, and it was in the university setting in the beginning, uh, for my masters, where I felt that, because of my, I guess, accent or, I don’t know, the way I speak English, that my- let’s say, my capacity was questioned. Eh. So, peers were surprised when I had, like, the best grades, um, and it was kind of very condescending the way, um, the whole thing was approached. Like, "Oh, I mean, how come she got the best paper?" (PS2, Venezuelan, 25 years, female, situational)  My German friends, because, like, 90% of my [friendship] groups are German people - um, like, I don't really understand how they- It's not that I don't understand, it's just that I cannot, like, feel the same towards their actions, towards something. You know, like, my culture is, like, very friendly and, like, warm, you wanna spend time together. And these people, when they don't wanna be, like, with you anymore, they were just like, “I'm tired, I'm going to sleep”. (PS3, Venezuelan, 23 years, female, situational)  It’s also when I came back to college [in the US], after being at home [in Poland] because of the pandemic. And I, like, I got back- I got back used- used to- back again, to-to being here, and to, like, the culture, to Poland, to the way my friends are, and, like, what’s cool, and what’s not cool, and, like, what we do as activities and stuff. And then I came back there, and everyone was different. And everyone would seem American or international, but both these groups were really, like, specific for me in how they behaved, in how they talked. (PS5, Polish, 29 years old, female, situational) |

**Table S2.4**

*Additional Quotes for Perceived Consequences of Not Fitting in With Social Norms*

| Positive consequences | Well, it has a positive consequence in a way. It helped me understand what I want and what I don't want. (T5, Turkish, 25 years, female, recurrent)  Let’s say, positive: in a way, I did develop, like, according to my own values, in a way. And I feel kind of proud of this, because I did not get influenced that much by social pressure in a way that I feel I had a very huge deviation to get actually back to meet myself and what I want. So, that is actually something that I would evaluate as something positive. (PS7, German, 28 years, female, recurrent)  And I feel like that also - like, on a positive note, I think that [being different from others] also attracts people. Cause there’s a lot of people that actually, like, uh deal with these specific feelings and they don’t really talk about it, like, quickly. But I talk about it quite - quite quickly with people. And I feel like that helps them, like, open up, and then that also helps p- helps building relationships quicker. (NL2, Dutch, 26 years, male, recurrent)  Yes, I’ve encountered different uh comments and - in actually positive ways of, like, ‘Wow, you have a beautiful energy.’ or, like, ‘You’re very different, you’re doing things in a very different way from what people are doing nowadays.’ Or, um… That - that’s where… I’ve - I’ve – I’ve really found very nice comments on that end, and um… (PS6, Venezuelan, 24 years, female, recurrent) |
| --- | --- |
| Discomfort | And um, like, I was in the hospital recently to get my tonsils removed and then my nurse, she was like ‘Oh yeah, is your family going to come and pick you up?’ Or, like, it to- ‘Is your family almost here?’, something [while participant has cut contact with his family]. She was already thinking that my family would come and pick me up. So, it’s already kind of like [participant makes noise to signal discomfort], you know? It’s, like, a bit awkward. (NL2, Dutch, 26 years, male, recurrent)  Yes, at the time I was one of the youngest in the team and that made me feel rather uncomfortable and, yes, I wasn't yet in a position to say anything about it [others tending to scream at people who had made a mistake]. So it was more a feeling of unease. (PS15, Austrian, 22 years, male, situational) |
| Frustration and anger | [When people commented on better not taking me on a work excursion because of my disability] I felt extremely aggressive. I couldn’t bring myself to slap them, but I did raise my hand. I couldn’t go through with it—only because we were standing in the office. […] I feel like you noticed—I have a lot of anger inside me. (D5, Indian, 27 years, male, recurrent)  Actually, there is anger and discomfort as well. Because when you express your opinion, you expect support or approval from people in your close circle, right? As I don’t receive that, there is anger and a feeling of lack of belonging - a bad feeling, and a feeling of being trapped. (T5, Turkish, 25 years, female, recurrent) |
| Not feeling understood by others | Despite having an accepting and safe friend environment, I think that they can understand me to a certain extent, but beyond that, I feel different. […] In my environment, everyone is straight and male, except for one female friend who is closest to me. Apart from her, everyone is a straight male. To a certain extent, they can understand me, and I can understand them up to a certain point. But beyond that point, it doesn't work anymore. (T1, Turkish, 28 years, male, recurrent)  And even with my closest friends, I sometimes feel like I'm not being heard and feel like I'm the odd one out in a group. […] And I shared this [a concrete incident where NL3 was agitated] with my friends, hoping to get some understanding or at least confirmation. But all I heard from everyone was, “Okay, you're being dramatic. Calm down, it's not that big of a deal.” And then I thought, “Oh guys, you're supposed to be my emotional safety net.” That's a shame. But then again, if you don't know how a mental disorder can affect your thought patterns and how that can make me feel emotionally, then I understand. (NL3, Dutch, 22 years, transgender male, recurrent) |
| Loneliness | Feeling different and lonely are kind of interwoven and all. […] So, I often feel like I am the only one in the world. It is not a good feeling at all. (NG3, Nigerian, 26 years, female, recurrent)  Where there is a difference, there is loneliness. All these things are connected. (D5, Indian, 27 years, male, recurrent)  [In an interview with Agnes Callard, a Hungarian-American philosopher, she] was talking about her marriage and her definition of loneliness was, uhm, the fact that you say something and aren't understood by others, and that really resonated with me because, like, I've always felt a bit weird and different. (PS12, US American/Belgian, 29 years, female, recurrent)  Nobody understands me, so I am always lonely. […] If I will explain loneliness, I will tell the child that; if the child has ever been in dark place or should I just say if the child has ever seen a crowd of people putting on black, and one single person putting on white in their midst; how different that person will be among everybody. (NG5, Nigerian, 24 years, male, recurrent) |
| Exclusion or rejection | One day, they all, my friends, we were, like, five or something, they were like, um, we're not gonna talk to you anymore until you start, like, talking like us. And I- that was like for two days, three days in school, like, they just didn't talk to me. (PS2, Venezuelan, 25 years, female, situational)  There are times that when they are having a discussion, they will say that I should not be considered in the discussion. […] At times, when they are having conversation and I passed by, they will just stop the discussion till I took my leave. Sometimes, they might say, “she's not part of the discussion, leave her alone”. (NG2, Nigerian, 23 years, female, recurrent but not throughout life) |
| Choices | I’ll not want to go to a club which is probably gonna make me feel uncomfortable. Or a bar that’s not really the setting that probably would be safe for, like, a gay guy to go to. (PS8, British, 22 years, male, recurrent)  That’s the major reason I left home and come over to Ibadan, so that I can be all of myself. (NG5, Nigerian, 24 years, male, recurrent)  Why bring up my disability? Why say, “Don’t let him walk,” or “That’s why we don’t want to take him with us”? That really hit me hard. I left the company immediately after that. I didn’t want to stay there anymore. (D5, Indian, 27 years, male, recurrent)  I also felt different at the start of my high school because I did some kind of hono- some kind of honors program where I did not really like the people because I felt they were boring. And then, after three years, I went into regular education because of this reason. (NL1, Dutch, 26 years, male, situational) |
| Sadness | Well, it makes me feel sad, because my thought was that they did not understand me very well. I was actually feeling sad and thinking that maybe things can get better than that. (NG6, Nigerian, 19 years, female, situational)  Well, like, I - the first time aware of this [being treated less nicely because of doing less well academically than what was expected], I still feel really, really hurt. And then I do feel like, ‘Oh, this is why sometimes, I just don't know why I'm not that happy, but I just know something goes wrong. (HK13, Chinese, 29 years, female, recurrent) |
| Anxiety and stress | It’s like my chapter doesn’t belong in their story. They speak so casually, without hesitation. And I keep wondering, should I say something about my identity? Is it safe? Will people judge me even in a professional space? (D8, Indian, 25 years, female, recurrent)  Um, I’m also gay. So, that was also a part of growing up where I did feel very alone. Um, and I remember the months before telling my parents and telling my friends and being so terrified of, like, what would happen and thinking ‘It’s just me. No one else must feel like this. It can’t be this hard.’ […] And that - and there’s that fear there of people weaponizing your sexuality against you. Um, and the fear of being victimized in the street or bullied by people or- or ostracized - pushed aside, definitely. (PS8, British, 22 years, male, recurrent)  Sometimes, I couldn't leave the bathroom out of fear, especially during breaks. […] I felt like I couldn't trust anyone, and if I came out, everyone would abandon me, and I would be left alone. Ultimately, the fear of loneliness was what I was avoiding. (T1, Turkish, 28 years, male, recurrent)  I had anxiety at some point. I would talk to people, turn around, and think, "Oops, I messed up somewhere." So, whether I was in that situation or not, it [feeling odd] would happen. (T10, Turkish, 26 years, male, recurrent) |
| Confusion and rumination | At times, I ponder more on what I could have to fit in, in this kind of gathering. So, when that feeling overwhelms me, at times, I ignore it. […] I just imagine what those people have that I don't. […] As I said earlier, I used to feel like "who are they that I am not." Those were the thoughts that came to my head. What are they doing that I am not doing right? (NG1, Nigerian, 30 years, male, recurrent)  I haven't—I still haven't figured it out [what made me different from other children so that they would exclude me]. […] Nothing explicit. I did try to get a neurological examination at one point, but they didn't find anything specific. […] it still bothers me, somehow. It's just strange that all the teachers and even the headmaster felt that it was my fault. […] And now, in hindsight, I understand that too, but at the time, I found it very confusing. (NL6, Dutch, 22 years, female, recurrent)  I have questioned why is beauty so important…and why me! Why did this happen to me! (D1, Indian, 21 years, female, recurrent) |
| Being signaled to be different or weird (e.g., through comments or jokes) | For example, people in high school used to always say that I was ‘weird’ or they knew me as ‘odd [participant's name]’ (T1, Turkish, 28 years, male, recurrent)  People thought I was “too serious”. Some people said I’m “too deep” or “too mature”. Not in a bad way, but in a “you don’t fit the vibe” way. (D9, Indian, 27 years, male, recurrent)  Yes, some people used to say I was a bit ‘off-beat’. Their tone wasn’t always supportive. (D7, Indian, 28 years, male, recurrent)  Like um, they notice that you think about it and then, when I do wear nail polish, they’ll, like, say something, but like ‘Oh, this is going on now.’ And then they don’t really think anything of it maybe, or they don’t say that - I’m not sure. But you do see that people notice things that are, like, different from the norm. Um, one patient, it was very funny. One patient was like, ‘You look like a clown’. Uh, he said that to me but, like, in a very joking way. (NL2, Dutch, 26 years, male, recurrent)  They sometimes called me the mother of Jesus Christ while some referred to me as “mommy GO” [GO = General Overseer, often used for a person who is perceived as overly religious]. […] Some could confront me and tell me that I am different. (NG2, Nigerian, 23 years, female, recurrent but not throughout life) |
| Disapproval or lack of acceptance by others; being signaled to be inferior or wrong | […] it’s always very specifically about, like, things that are a bit more, like, femme presenting. So, it’s very apparent that, like, what- what is it that they don’t like. So, then it - that makes it hurtful. Um, and also, like, them, like questioning it as if they think that I didn’t think - didn’t think it through. So, like, there’s a very, like, top-down thing there as well. Like, ‘Oh, like, you obviously didn’t think this through. So, I will tell you that it’s weird. So, you know that it’s weird. And then you will probably change it.’ As if they already know beforehand that they’re, like, smarter than me. Or that they know more about the world than me. (NL2, Dutch, 26 years, male, recurrent)  And once, like, I remember, like, one of my friends’ friend… Yeah, he was like, “Yeah, she doesn't do drugs.” And he was like, “What the fuck? That is not cool.” And I wa- then, I was like… Just kind of laughed and I was like, “Are you seriously saying that I'm not cool because I don't take drugs?” And he was like, “No, I'm kidding.” And I was like, “Are you?” (PS3, Venezuelan, 23 years, female, situational)  So, I was different in type of - this interest and then you’re uncool right away or at least I was afraid that they see me either as a nerd or uncool, although I was not. (PS7, German, 28 years, female, recurrent)  They include me, but they treat me very shamefully. (D4, Indian, 30 years, male, recurrent) |
| Being misperceived or stereotyped | Sometimes, people have different opinions about me, like I am difficult, I am not approachable and all that. (NG1, Nigerian, 30 years, male, recurrent)  Mmh, I think so. I think I come across as being, uhm, very, like, happy and smiley and kind of like, not stupid, but kind of like, naive and like, kind of cute in a stupid way. (PS12, US American/Belgian, 29 years, female, recurrent)  In fact, they might perceive my preference for not communicating with them as a somewhat condescending attitude. […] I can notice it from their attitudes. My feeling that I don’t belong is reflected by them. It makes them think that I think that I'm better than them. (T5, Turkish, 25 years, female, recurrent) |
| Inferiority, insecurity, or self-monitoring | I don't know, but it brings down my self-esteem. [...] Just loss of self-confidence, feeling bad, and having most of the time negative thoughts about myself. […] Also, my self-esteem is hitting the rock in recent times. Situations that might have allowed me to meet new people or make more friends is forfeited, because of the fear of feeling differently. Even, when someone tries to initiate friendship or conversation with me, I will just cut off from the person, because at the end of the day, the person might still leave or ignore me. (NG3, Nigerian, 26 years, female, recurrent)  Well, yes, my elementary school experience contributed greatly to that. I feel that it caused me to develop a kind of lasting fear that there is something about me that everyone else can see except me. Something that makes me different. Because it was very specific that I, and I think one other child, were excluded in class for six years. […] So, that created a very strong feeling that there was something wrong with me, but I didn't know what. […] I really wanted to get rid of it – that others would notice it. […] [It resulted in] A lot of insecurity and shame, especially. […] Um, yes, but also a lot of, um, insecurity and shyness. Um, in elementary school I had a really hard time with—still do—my voice was very soft and I heard “can you speak louder?” every 5 seconds. Um, and I just couldn't do that very well. And now, I notice that occasionally, when I'm in a new situation that I’m nervous about, it happens again. My voice is still not—it doesn't go very loud, actually. (NL6, Dutch, 22 years, female, recurrent)  Yeah, I think – I think this kind of, like, feeling of not being good enough or might being rejected is something that I - I do take with me to other experiences as well. Like, for example, when I’m dating, I… There’s like this core cognition - thought of, like, lacking, or not being good enough or people not, like, appreciating me for who I am. So, I have to change who I am to be appreciated. […] During studies, I went through, like, this period just, like, thinking as well, like, ‘I’m not good enough and I can’t finish this, like, in a way that I should.’ Cause that’s, like, the thought that was instilled in my brain. Uh, so, then I, like - I did, like, quit studies a couple of times or I took breaks. (NL2, Dutch, 26 years, male, recurrent) |
| Conflict in relationships | Sometimes that causes conflict with them [others]. Cause I don’t influence my ideology on them, but they don’t do the same! They force it upon me... to be like that. That leads to conflict and I just leave the conversation then. (D4, Indian, 30 years, male, recurrent)  And I just felt really bad about myself, which meant my parents didn't really know what to do with me. And when I got a little older, I started—that was more around high school age—I started to really lash out, so to speak, and act out a bit. Um, that was just me—well, I was quite a depressed teenager and I wasn't very sociable, let's say. Um, and my whole family just didn't really know what to do with me, and we had quite a tense family dynamic. Um, yes. Which also caused my parents and my little brother to sometimes gang up on me. Like, yeah, what's going on? We don't understand her. (NL6, Dutch, 22 years, female, recurrent)  I discovered this with my therapist; I can share it if you want. She talked about a perspective. Like, she said, "Your older sister, how much older is she than you?" I said, "9 years older." “The other one, how much older is she?" "12 years older." “Your parents are 30 years older. So, you have a group, and you joined it later.”, she said. This was a kind of enlightenment for me; I accepted it as such. I wonder if my feeling of being disconnected from these groups throughout my life is because I was born that way, in that state of mind, and turning it into a competition there - like the feeling that I have to be here, I have to exist [survive / stand my ground] here? Is that why, in certain periods of my life, with certain groups of friends, I'm always getting into these conflicts? I try to exist [survive / stand my ground], and then I aggressively throw myself out there. (T3, Turkish, 26 years, male, situational) |
| Not being able to join conversations or social activities | When I'm with them in a group setting, there are times when I can't find the right words to contribute to the conversation. (T5, Turkish, 25 years, female, recurrent)  Who's getting an arranged marriage, who’s going for love marriage, honeymoon plans, they were even discussing baby names. I stayed quiet. (D8, Indian, 25 years, female, recurrent)  And, like, you see your friends – um, like prom, like, going to prom at school. Like, seeing the boys ask girls to prom and me just being like, ‘I –‘ - because obviously it was very special for them, um, as, like, heterosexual people. It was kind of, like, cute and romantic. But for me, it was very much ‘I can’t really have that’ because there’s not - I can’t really approach one of the lads at– well, especially not the school that I went to. There was no way that I could approach one of the guys and be like ‘Do you wanna go to prom?’, you know? […] Um, and obviously being different there, then that makes you feel lonely. Because you– you can’t really get out of that experience what everyone else is getting out of. Obviously, you would probably enjoy it but not to the same level as what other people would. (PS8, British, 22 years, male, recurrent) |
| Difficulty finding or retaining relationships | This feeling of difference has not made me keep any friend for up to two years. The longest I have had a new friend is like a year plus. Just a year plus; and after a year, it is gone. The only friend I have presently is one, is still my friend by being persistent about keeping the friendship. I have been the one calling to keep our line of communication open. But others, after one year pass, bond is already reducing; and all of a sudden, we become strangers again. (NG5, Nigerian, 24 years, male, recurrent)  Also, I don't have many friends (in the class), I only know them by name. I haven't been able to establish a close connection with anyone. (T6, Turkish, 26 years, female, recurrent)  […] there are already so many things in my life that are missing [due to living with disability]. I can’t even ride a scooter. I can’t drive a car. I don’t have a partner. I don’t have a love life. And honestly, I don’t think it’ll ever happen. (D5, Indian, 27 years, male, recurrent)  And then also, as I feel like dating - queer dating is quite difficult. Um, and then there’s people around you that have, like, children and families and… (NL2, Dutch, 26 years, male, recurrent)  [To me, it seems easier to] get into a heterosexual relationship, because like - I feel, on a night out, you can usually tell when a guy is straight and a – and a g- and like, a girl is straight. And when they usually like talking to each other, I feel like you get chemistry. But with - I don’t know if it’s just me, but talking to s- like, some guys, you don’t, like, you don’t know - you really don’t know what’s going off. And I feel like – again, it might just be my experience, but I feel like heterosexual people can read each other’s, like, attraction, a little bit better. Sometimes it’s a little bit harder for homosexual men, in my opinion. […] Um, and so, definitely the anger, because you see other people getting on in relationships so well in heterosexual couples because it’s so normal (PS8, British, 22 years, male, recurrent) |
| Verbal or physical aggression | I mean, I did experience some bullying in high school. […] from upper-grade students, yes, I felt it a lot. Sometimes, I couldn't leave the bathroom out of fear, especially during breaks. […] It was more about psychological violence based on my appearance. There wasn't much physical stuff but escaping from psychological violence. (T1, Turkish, 28 years, male, recurrent)  So, the thing is that in school, there was a bullying issue in the beginning. When there was bullying, self-confidence was lost at that time. (D5, Indian, 27 years, male, recurrent)  There’s that fear there of people weaponizing your sexuality against you. Um, and the fear of being victimized in the street or bullied by people or- or ostracized - pushed aside, definitely. (PS8, British, 22 years, male, recurrent) |
| Non-belonging | That's why I feel like I don't belong there and I don't feel a strong sense of connection. […] Actually, there is anger and discomfort as well. Because when you express your opinion, you expect support or approval from people in your close circle, right? As I don’t receive that, there is anger and a feeling of lack of belonging - a bad feeling, and a feeling of being trapped. (T5, Turkish, 25 years, female, recurrent)  Maybe that moment made me feel that I don’t belong anywhere. (D3, Indian, 20 years, female, recurrent) |

**Table S2.5**

*Additional Quotes for Coping Strategies and Suggestions for Interventions*

| Leave social context where one does not fit in; change social contexts | Um, and now, let’s say, as soon as I started my studies or as- as soon as I finished high school, I really decided to explore the world and just get away from here, basically, and say: “Okay, let’s see, maybe it’s not me being weird. Maybe I just don’t fit in. So, let’s figure out, maybe there are other places.” Also, I have an older sister. She also travelled a lot and she lived in different places and I really saw that she might – like, she feels better somewhere else. So, I was like, “Maybe it works for me as well in a way.” (PS7, German, 28 years, female, recurrent)  When I know that where I am is not in line with my moral side; I tend to just leave; even without them asking me to leave. I will excuse myself just like I did few days ago. (NG2, Nigerian, 23 years, female, recurrent but not throughout life)  Um, I think it's more that I try to avoid these situations, where I already have the suspicion that people might be different to me. So, um, yeah, maybe, like, being less likely or being a little bit less open-minded towards, um, like, specific meetings, specific people who are doing specific activities. Especially when they're in a context where I have the, like, idea that people who usually do these kinds of activities are very different from me. Then I would try to avoid this. (PS4, German, 26 years, female) |
| --- | --- |
| Fitting, accepting, or understanding relationships or community | When I met people like me — what we call "our tribe." Connecting with online creative groups and a few real-life friends helped too. (D7, Indian, 28 years, male, recurrent)  Most of the new friends I made after graduating came from fandom [following celebrities and traveling to see them]. Whenever I traveled for events, I’d meet people who shared my interests. Sometimes we’d meet up in person again at other events, and other times we’d just chat online. But our conversations weren’t only about fandom. We’d talk about our studies, our hobbies, our lives—not really about family. (HK8, Chinese, 24 years, female, recurrent but not throughout life)  However, when I see people of like minds, I am always happy. It is just like Prophet Elijah in the Bible thought he was alone, but when God told him that he has over 7,000 or 700 prophets that have not bowed down their heads to baal. So I am always grateful to God that I am not alone in this Christian journey. (NG2, Nigerian, 23 years, female, recurrent but not throughout life)  I was almost twelve when I got Minecraft, because I had been asking my father for six months to buy me the game. And then finally, after a lot of persuasion, saying, “Yes, but my friends have it too.”, he finally bought it for me. And then I was online – there was an online server where I could talk to other people, and I started talking to them a lot. After six months, we had a group chat with a few people from that server who I hung out with a lot. And they were very – I was the youngest in that group. I'm still the youngest in the group. Yes, um, but those were people I could really talk to. I also noticed that they were a bit more on my level than many of my classmates in Year 8, for example. Because, for some reason, I was a bit further along in my cognitive development. So there - with those people I could talk a lot - very v- uh, very nice conversations. We had the same interests, because we were all playing the same game. They were all doing the same thing. And then - when I was about twelve, thirteen, that group really started to become my regular group. And I really liked that because finally, for the first time in my 12, 13 years, I finally had a regular group of friends with people I could really talk to. (NL4, Dutch, 21 years, non-binary, recurrent)  But then I found my core group—my friends in master’s. With them, I never felt different. They accepted me. (D9, Indian, 27 years, male, recurrent)  Yeah. I think for me, personally, like, the fact that I met people that I have so much in common with, that understand me so well, that are also queer or understanding of the queer experience, I think that has been a massive uh thing to, like, make things better for me. To have this support network means a lot to me, personally. And that’s something that I built most of my things around, yeah. (NL2, Dutch, 26 years, male, recurrent)  When I joined an LGBTQ+ art collective, for the first time I felt like my identity could be celebrated. Everyone there had unique experiences, but we all felt connected. That space taught me how to belong. (D8, Indian, 25 years, female, recurrent) |
| Accepting, supporting, and relying on oneself | So, I’ve made peace with simply feeling that I am my own person and I am – I am who I am. I have and hold the beliefs that I do, and whether I fit or not, that’s really not my problem. […] I am who I am. As long as I don’t hurt anyone, I am being myself. (PS6, Venezuelan, 24 years, female, recurrent) |
| Acceptance of not fitting in; less sensitivity to being different from others | It is the joy. The joy of the Lord is my strength sincerely. There are times that I would want to think about the way that those people act, that is why I cannot act same way. But, the joy will just come. I am different. Other people may be doing this, but then I am not for other people. (NG2, Nigerian, 23 years, female, recurrent but not throughout life)  And like, I’m starting to learn that people that are different to - to me can also be nice people that wa- want to understand. One patient was like, ‘You look like a clown’. Uh, he said that to me but, like, in a very joking way. But I was like - and I was like ‘Yeah, I completely agree. Like, I - sometimes I look very clownish.’ […] And then on the other side, which I think is kind of like coping, is I feel very much like, ‘It’s fine. This is just how it is.’ (NL2, Dutch, 26 years, male, recurrent) |
| Trusting in time reducing feelings of difference | Now I have made peace with it, as I know that it takes time to get better and I am working on it. (D3, Indian, 20 years, female, recurrent)  To reduce these feelings - well, I just left it to time, and I convinced myself a bit with my own thoughts. (T3, Turkish, 26 years, male, situational) |
| Distraction from negative feelings | *Engaging in enjoyable activities:*  When you spend your time invested in something you are good in. So, when I do that, that is how it goes away. (NG1, Nigerian, 30 years, male, recurrent)  And that you then simply do things for yourself that interest you and also enjoy this time alone in order to gather new material, new influences, new experiences, so that you can share them again, because that in turn provides points of contact [for connection with others]. (PS14, Austrian, 27 years, female, recurrent)  Yeah, in that moment, doing yoga helps. Yoga really helps. It makes me feel good. […] Sometimes I go for a walk. (T2, Turkish, 26 years, female, recurrent)  That's still following celebrities. […] Yeah, after I quit my job and graduated, I started traveling to different places. […] it was always for fandom. I’d travel to Hong Kong, Macau, and other places to see my favorite artists. […] Most of the new friends I made after graduating came from fandom. Whenever I traveled for events, I’d meet people who shared my interests. (HK8, Chinese, 24 years, female, recurrent but not throughout life)  *Engaging with media:*  You know, I said, when I'm with my phone, my phone is my best friend. So, when I'm with my phone, I just take my phone and then press it. (NG10, Nigerian, 19 years, female, situational)  These days, I no longer converse with others. I just engage my books, watch movies, play games. […] But thanks to God for social media, once I get engaged with my phone and watch movies, I just get my mind off it. […] There are apps on my phone that keep me engaged. (NG3, Nigerian, 26 years, female, recurrent)  I prefer listening to songs on YouTube, songs that I like. Or I watch poetry on YouTube. (D5, Indian, 27 years, male, recurrent)  My own solution is just a distraction. Anything like social media that I could use to while away time or by watching movies. (NG1, Nigerian, 30 years, male, recurrent)  My phone. It is either I start watching a movie or I start listening to music, or chatting; by just going through social media. (NG2, Nigerian, 23 years, female, recurrent but not throughout life) |
| Talk about experiences of not fitting in | What makes me feel better is sharing this situation with my own friends. Sometimes, when I feel so detached, to the point where I question if I'm the abnormal one in this situation, I share this with friends who are on the same wavelength as me. And because they think similarly, this provides some relief. (T5, Turkish, 25 years, female, recurrent)  And I have few people that I can, um… that I really connect with and when I do have, um, issues or things that I would like to talk about, I- I- I give them a call and - and then I just talk things through and that’s the way that has helped me to- to overcome things. To just have that support group uh that’s- that’s there. […] So, I think those are the people that, in those moments, uh, we should reach out. Just to see, you know, contrast if - sometimes you think you’re crazy, but no, it’s not you, it’s uh - it’s- it’s– it’s in your head, like whatever label you’re putting for feeling x or y, it’s- you’re not crazy. (PS6, Venezuelan, 24 years, female, recurrent)  I think sharing definitely really helps. […] I guess I just mean that, uhm, if I share with someone that I really know and that I feel more close to, then maybe I'm going to feel, like, more understood and safer. (PS12, US American/Belgian, 29 years, female, recurrent) |
| Hiding deviation or part of oneself; not opening up | I was being careful to really open up, because I just couldn't relate very well. (NG7, Nigerian, 25 years, male, recurrent)  I still remember, like, in, in math class in high school I, I would finish the exercise like really quickly and then I'd pretend to still be working so that people wouldn't think that I was already done, uhm... (PS12, US American/Belgian, 29 years, female, recurrent)  *Being in the closet:*  I’d probably say no cause I - I used to mask – like, mask who I really was really well. And I think I did it for a really long time. […] So, my first ever proper relationship was six months with a girl. And that was, I think, the result of feeling different [for being gay] and sort bu- sort of wanting to, like, hide it. Um, and obviously, that was me just trying to – again, keep it away from everyone and having to deal with the truth and tell people. (PS8, British, 22 years, male, recurrent)  I remember this one incident! Everyone was chatting during lunch about weddings. Who's getting an arranged marriage, who’s going for love marriage, honeymoon plans, they were even discussing baby names. I stayed quiet. I was smiling, but deep inside I felt completely cut off. I thought, if I said I had been in a relationship with a woman, the entire atmosphere would freeze. […] Yes, there’s a vivid memory from school. After 11th-grade summer break, all the girls were talking about their crushes and boyfriends. I liked a girl, but I couldn’t say anything. When someone asked me who I liked, I lied... Just named some actor. Deep inside, it felt like I was cheating myself. At that age, confusion is normal, but when your truth feels unacceptable, you start drifting away from yourself. (D8, Indian, 25 years, female, recurrent)  I think, they delayed the process of accepting my gay identity and coming out to people. It made me think that perhaps everyone is homophobic, at least to some extent. This, in a way, made me more cautious about revealing my true self. Socially, it made me feel like I needed to be more guarded. (T1, Turkish, 28 years, male, recurrent) |
| Disengaging or becoming uninvolved | Even though they tried to include me. I didn’t wanted to as I felt that I had to talk in a certain way. […] I was just agreeing with everything they were saying. And I was like everything is okay and yeah. It’s good. (D3, Indian, 20 years, female, recurrent)  I know that they don’t understand different ideologies or idea, so after that, it becomes difficult to share any ideas with them. So, you are like ‘It’s waste of energy with them’. I feel like ‘yaar (=man), why am I even discussing with them?’ (D4, Indian, 30 years, male, recurrent)  Um, it made me feel a bit silent in a way. It- I didn’t really feel anger back then. I felt rather, ‘I don’t even wanna be part of it.’ So, I was rather turning away instead of, like- instead of trying my best and feeling anger, I was like, “No…” Like, you know, this silent decision: watching, observing. I was always observing people. I was like, ‘Do I wanna be like them? Maybe no. No. I don’t wanna do bad to other people, I don’t wanna have a bitchy behavior, um, this short lived girl-/ boyfriend thingies…’ (PS7, German, 28 years, female, recurrent)  It could be during some conversations when I brought up topics I wanted to discuss, but I found that their opinions were different from mine. […] And then that would stop me from going deeper or opening up more about myself. (HK3, Chinese, 24 years, female, recurrent) |
| Social withdrawal (as negative consequence) | I sticked [sic] mainly to myself. I did have some friends, like very loose friends, but very shallow friendships. So, I didn’t really feel comfortable around them. That was kind of a common thing. So, rather stick to myself and did my own thing. […] Even now at university, I try to be neutral with everybody. And back then, at my- in my- during my bachelor’s and master’s as well. So, [this experience of not fitting in at school] kind of makes me even now this loner. So, it was mainly people in school. I had friends. Apparently they liked me because they keep invi- kept inviting me over for, yeah, hanging out and stuff. I sometimes went, but I always- I remember I didn’t really wanna go. […] [Due to] Anxiety of judgement of other people towards me. So, with myself, I felt fine. And that goes on until today, but I’m afraid of judgement of others towards me. (PS7, German, 28 years, female, recurrent)  I was with myself a lot. Like, I was in my head a lot and I was in my room a lot. Um, that’s how I remember that. […] Yeah, um, I think it has two sides. I think on the one hand, I do have a tendency to isolate myself sometimes cause I feel like I should, like, just all solve it myself. Cause I can’t, like, trust the outside world anyway. So, I should have to fix it myself. (NL2, Dutch, 26 years, male, recurrent)  One is simply isolating yourself and vegetating away in sadness, so to speak. That's not a decision, but it's just this increasing isolation that has also triggered a certain, yes, something negative, a lot of negativity, which then intensifies the other things, of course. (PS14, Austrian, 27 years, female, recurrent)  Then, I don't know what caused it, I just know that I want to withdraw. I do not want anybody around. One time, I might want people around, while some other time, I may not want them around. It's going and coming like that. […] (NG5, Nigerian, 24 years, male, recurrent) |
| Social withdrawal as coping strategy | It definitely helps. In fact, I can say that I can resolve most issues, like the issue of loneliness, by being alone. I can't resolve them when I'm among people, because I lose control at that moment. (T3, Turkish, 26 years, male, situational)  So, most time, I just walk on my own in order to free my mind. Not that I'm holding grudges against that person, but that's just the person I am. […] I'm okay on my own. (NG9, Nigerian, 21 years, female, situational)  Um, and then Covid happened. And although it was awful because obviously so many people died and became really poorly, for me, sort of being trapped and not being able to go out was kind of nice in a way. Because I was able to come to terms with what had happened over the past few years to sort of understand how I really felt. Um, so, I- I think I definitely didn’t feel as alone [because of having made different experiences than other and struggling with mental health more] after that. (PS8, British, 22 years, male, recurrent) |
| Try to adapt or conform | *Negative perception of attempts to conform:*  But, um, let’s say, if I pretend that I like stuff that I don’t like - let’s say, in the - in the bigger picture, it might also lead me to people that are actually - that are - that are not a match, which will not support my mental health. (PS7, German, 28 years, female, recurrent)  So, I’ve actually made peace with the fact that, yes, I don’t need to fit because, even if I try, I am trying, so that’s not me. So, if I have to try something, then I am trying to fit. That means, I don’t feel good with who I am. And if I - And I’m - As I said, ultimately, you’re not yourself. (PS6, Venezuelan, 24 years, female, recurrent)  I tried to engage in conversation with them, like talk to them. So, I do what I see others do. So, I don't know why I am being treated differently. (NG3, Nigerian, 26 years, female, recurrent)  Uhm, and I always felt like, I was weird and I, I would try to, like, act like others, but it didn't quite work. (PS12, US American/Belgian, 29 years, female, recurrent)  *Positive perception of attempts to conform:*  I can easily adapt to the environment I'm in, so I haven't experienced that feeling [of not fitting in] much. (T8, Turkish, 19 years, female, no experiences of feeling different)  When interacting with people, I almost put myself aside. […] It means not imposing my stance and opinions. […] Getting along with this roommate, I just go along with what he wants to do. If he wants to do something, I accompany him, yes…… […] It's not a feeling of accommodating. Because if you are accommodating someone, you would feel tired, but I don't feel tired. For example, in the Dao De Jing, there's a concept of water, right? You know, water is fluid, it changes, it's that kind of feeling. (HK2, Chinese, 19 years, male, recurrent) |
| ***Possible interventions*** | |
| Support groups | Yes, that's the whole reason I had to go to group therapy. Because I started with individual psychotherapy and schema therapy in connection with personality issues. And at some point, my therapist realized that I still had the idea that—even though, if I look at it logically, that's bullshit, of course—I still had the idea that I was the only person in the world who experienced these kinds of things and reacted to them in this way. And that's why I started with that group, just so I could see what I was going through, that I'm not alone in this. And okay, not everyone has experienced exactly the same thing as me. But people recognize that feeling, and you're not the only one. (NL3, Dutch, 22 years, transgender male, recurrent)  There are many people feeling the same way in different contexts, so why not come together, share, and it's a known fact that if we share all this, it feels a lot lighter. (GJ1, Indian, female, situational)  So, it's like, maybe if there was, like, some kind of support group - maybe not, like, branded as a support group, but just, like, a group, like, “let it all out” or something. Yeah, it's a support group, but I think it's, like, just a support group thing, it's, like, kind of, like, a confronting thing. You- you know, you're just like, “Is it something wrong with me?” or like, “Is it people there that are not doing so good?” You know what I mean? (PS3, Venezuelan, 23 years, female, situational)  Support groups can be really helpful. Even in workplaces, there should be mental health or inclusion circles that provide a safe space. (D7, Indian, 28 years, male, recurrent)  I'm not sure about the context of a support group. […] I went to this, like, sort of group several, a couple years ago for people with, like, different neurological profiles, and, I don't know, I feel like, when it's people you don't know, it often kind of, like, one person monopolises the conversation, one person has their own agenda of what they want to talk about. Uhm, so, I think sharing is really, really helpful but I think for me personally, I need to be careful about who I share with, I think. […] I don't know, but maybe support groups would be helpful. I, I've never, apart from that one time, I've never really tried, except this one thing that I did […] and it was interesting to hear about everyone's experiences. And I guess that does make you feel less different or less weird or less lonely. […] So, maybe it could help. (PS2, Venezuelan, 25 years, female, situational) |
| Increase tolerance and acceptance of diversity in society | But it's usually just an institutional problem, which isn't explained to children: “Hey, some people look a little different or some people think a little differently. That's not a problem. They just do different things. Just like apples don't make pears. There's nothing wrong with that, it's just different and that's okay. And if I had known that, and if my peers or my friends had known that, and if it had been clear to everyone, I think it would have been much nicer. Just like now, for example, you see more and more attention being paid to people who are in wheelchairs or people who have difficulty walking, so that they can also be accommodated. But we all know that some people can do this, some people can't, or some people like this or don't like it. (NL4, Dutch, 21 years, non-binary, recurrent)  I think if you want to correct stereotypes, it takes a long time, it's all about subconsciously influencing the students little by little, telling them slowly, and correcting these stereotypes over a long period of time by education. So, I was thinking that maybe our generation is already like this, and then maybe those who can reflect on it will think about it more and then make themselves change. And then focusing on the children's generation, letting them grow up in an environment where they're not constantly being told that boys are good at math and girls are good at languages, and that boys go to play soccer and then girls go to play handkerchief throwing and things like that, and not defining that for the children, and letting them grow up in an environment where they're less stereotyped. (HK7, Chinese, 28 years, female, situational)  Yes, schools should have identity education where every student feels safe and heard. In workplaces, diversity shouldn't just be a checkbox, it should mean real inclusion. (D8, Indian, 25 years, female, recurrent)  But in many ways, this is systemic, like- So, why is it that a person has to be responsible for confronting the challenges of discrimination, when, in the first place, discri- discrimination shouldn't be there. So this whole kind of fight, I guess, between adapting to a situation and changing the situation and whether there is agency in that person to change the situation, I guess that's where I think real change is needed. (PS2, Venezuelan, 25 years, female, situational) |
| Professional help | [I do not feel different from others] too often now since my interactions have gotten better. All thanks to therapy! I am thankful! (D6, Indian, 28 years, female, recurrent)  They [people who feel different] could definitely go to therapy to understand the dynamics of not feeling belonging to that environment, why they feel that way, and so on. Also, there's a reason why feeling that one does not belong causes discomfort. Feeling like one does not belong to a place can sometimes be beneficial. So, why does it feel bad? Why does this person feel bad? Maybe a [therapy] plan can be put together here: Understanding the reason for feeling bad and creating a plan for feeling better. (T9, Turkish, 27 years, female, recurrent but not throughout life)  I'm not a psychologist, but I know that some psychological principles do help. I think the help psychologist could actually render might be temporal. (NG7, Nigerian, 25 years, male, recurrent) |
| ***Cultural differences in coping strategies*** | |
| Turning to religion and faith | And if there was anything that helped me from that situation, because it was like a turning point for me; was that I actually had an assurance that if nobody cares, God Himself cares for me. That was the only thing that saved me from those situation. […] in all of these things, there's this assurance I always have within me that, even when nobody cares, the Lord cares and he understands me. That I have someone that understand the situation much more than anyone else. That gave and still gives me confidence. (NG7, Nigerian, 25 years, male, recurrent)  God should just help me as a Christian. […] It is just like Prophet Elijah in the Bible thought he was alone, but when God told him that he has over 7,000 or 700 prophets that have not bowed down their heads to baal. So I am always grateful to God that I am not alone in this Christian journey. […] It is the joy. The joy of the Lord is my strength sincerely. There are times that I would want to think about the way that those people act, that is why I cannot act same way. But, the joy will just come. I am different. Other people may be doing this, but then I am not for other people. […] Ah, what can help me is the Holy Spirit; that, even when I see that I am different, I should be able to hold onto the standard. (NG2, Nigerian, 23 years, female, recurrent but not throughout life) |
| Letting go of ego / self | Like, why people react to certain things or not and, like - and that has to do a lot with the ego. […] So, in that way, I do feel that having some type of a different sight has given me a different, um… has given me tolerance and understanding. (PS6, Venezuelan, 24 years, female, recurrent) |
| Distraction through phones | For quotes, see *Distraction from negative feelings* on p. 33. |
